# Generation of spatiotemporal optical coil with controllable transverse intensity chirality

**DOI:** 10.1038/s41377-026-02432-0

**Published:** 2026-11-09

**Authors:** Dana Jiashaner, Xingyuan Zhang, Huiming Wang, Zhirong Tao, Qian Cao, Dawei Liu, Daijun Luo, Lu Yang, Kaili Geng, Yonghang Tai, Xinhua Xie, Zhensheng Tao, Md Sabbir Ahsan, Guangyu Fan, Qiwen Zhan

**Affiliations:** 1 School of Optical-Electrical and Computer Engineering, University of Shanghai for Science and Technology, Shanghai, 200093 China; 2 Zhejiang Key Laboratory of 3D Micro/Nano Fabrication and Characterization, Department of Electronic and Information Engineering, School of Engineering, Westlake University, Hangzhou, Zhejiang 310030 China; 3 Shanghai Jiao Tong University, Shanghai, 201100 China; 4 Karlsruhe Institute of Technology, Karlsruhe, Germany; 5 Department of Physics and Electronic, Yunnan Normal University, Kunming, Yunnan 650000 China; 6 Photon Science Center, Paul Scherrer Institute, 5232 Villigen PSI, Würenlingen, Switzerland; 7 State Key Laboratory of Surface Physics and Department of Physics, Fudan University, Shanghai, 200433 China; 8 Fachbereich Physik, Freie Universität Berlin, Arnimallee 14, 14195 Berlin, Germany; 9 International Institute for Sustainability with Knotted Chiral Meta Matter (WPI-SKCM2), Hiroshima University, Higashihiroshima, Hiroshima 739-8526 Japan

**Keywords:** Ultrafast photonics, Nonlinear optics

## Abstract

Chirality is a fundamental asymmetry in nature. In optics, control of chirality is evolving from spatial properties like polarization to the temporal chirality of local fields. Conventional approaches, however, encode chirality in abstract degrees of freedom such as phase or polarization, limiting direct and efficient coupling with matter on ultrafast timescales. We introduce and experimentally demonstrate a paradigm of intensity-driven optical chirality, where handedness is physically inscribed into the trajectory of spatiotemporal energy flow. Through the engineered non-collinear collision of Spatiotemporal Optical Vortices, the spatiotemporal optical coil is generated. The coil’s chirality manifests as a physical torsion of its volumetric energy flow, a property distinct from chirality hidden in phase or polarization. By employing femtosecond tomography and phase-resolved reconstruction, we map the complete three-dimensional spatiotemporal intensity field, directly observing the evolution of singularity lines. This approach reveals programmable scaling relations between the coil’s topology and interaction geometry, extending the concept of chirality to the energy-flow level. The introduced volumetric chiral density directly correlates with the optical forces and torques exerted on chiral particles, providing an intensity-manifested degree of freedom for topological photonics and enabling novel schemes for ultrafast chiral-selective excitation and high-dimensional information encoding.

**Figure:**
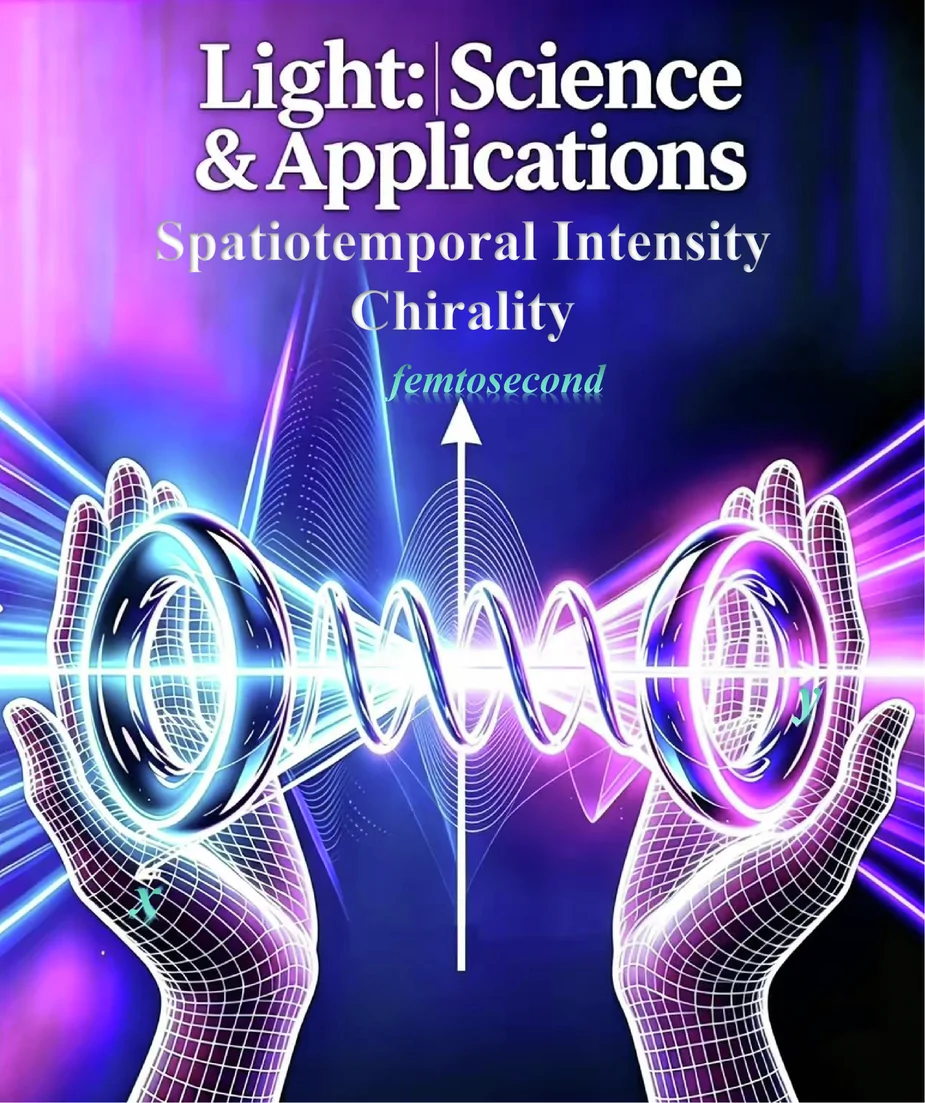

## Introduction

Chirality manifests across diverse physical phenomena spanning multiple scales in nature: from molecular handedness in amino acids and saccharides to the conformations of biological macromolecules^1,2^, and further to helical galaxies at cosmic scales, atmospheric vortices^3,4^, as well as chiral spin textures and vortex reconfigurations in condensed matter^5–9^, and in fluids^10,11^. Beyond serving as a geometric descriptor, chirality profoundly influences selectivity in mechanical, optical, and chemical processes. Conventional chiroptical detection methods, such as circular dichroism, rely on the electric dipole-magnetic dipole interference effect in light-matter interactions. However, when the wavelength of light far exceeds the molecular scale, the resulting chiroptical response is exceedingly weak, posing a significant challenge to high-sensitivity, high-throughput detection^12^. Recently, optical chirality research has entered a new era. A breakthrough concept envisioned by Smirnova redefines chirality as a local, time-evolving property of a three-dimensional vector trajectory^13^. This paradigm has given rise to new concepts such as local chiral light and temporal chirality, which dramatically enhance the efficiency of light-chiral matter interactions by engineering optical fields to be chiral in the time domain rather than the spatial domain. Methods based on this principle, including photoelectron circular dichroism^14^ and nonlinear frequency conversion schemes^15^, have elevated the sensitivity and speed of chiral recognition to unprecedented levels. This also opens avenues for addressing fundamental scientific questions, such as the origin of cosmic homochirality and parity-violating energy splitting^16^. Crucially, this perspective pushes the research frontier toward the interface of chirality and topology, heralding the possibility of robust measurements of chiroptical signals using topological invariants.

Most approaches to manipulating chirality in optics rely on abstract degrees of freedom such as polarization or phase winding. In contrast, we explore a paradigm where chirality is encoded into the volumetric spatial distribution of field energy density. While intrinsically linked to phase, this approach manifests mirror asymmetry explicitly through twisted configurations of the intensity envelope. This approach establishes mirror asymmetry through twisted configurations of electromagnetic energy in space, enabling direct chirality-specific responses in otherwise achiral objects, exerting optical forces and torques, and biasing nonlinear optical processes. Recent advances in sculpting electron density into three-dimensional helical structures (Electron Coils)^17^ within free-electron systems further underscore that intensity- or density-encoded chirality is a universal concept spanning diverse physical systems. While such static manifestations facilitate device integration and steady-state coupling^18^, they inherently constrain direct investigations into the temporal dynamics of chirality generation, propagation, reconnection, and dissipation^19,20^. Moreover, they impede the exploration of coupling mechanisms across different scales and systems, limiting our ability to probe the ultrafast evolution and transformative interactions of chiral phenomena in dynamically evolving settings.

To address this gap, we propose and demonstrate a novel approach based on programmable spatiotemporal vortex wave packets. Recent advances in sculpted spatiotemporal optical fields^21–25^—such as spatiotemporal optical vortices (STOVs)^26–28^, Optical Tornadoes^29^, Optical Skyrmions^30^, Hopfions^31,32^, Torus Knots^33,34^, and the newly identified Optical Rotatum^35^—have extended singular optics into the full spatiotemporal domain^36–38^, revealing rich topological and dynamical phenomena. It is important to note, however, that existing studies have largely emphasized the role of phase or polarization in driving chiral material responses, yet the underlying physical mechanisms through which such fields impart chirality-specific forces remain incompletely understood, thereby limiting broader applicability^39^. In this paper, we generate the spatiotemporal optical coil (STOC) via programmed non-collinear collisions of STOVs. As summarized in Table 1, structured chiral light can be categorized into three distinct types: the first type based on spatial polarization/phase, the second based on temporal chirality, and the third—spatiotemporal intensity-encoded chirality—realized in this work through the three-dimensional spatiotemporal helix of energy flow. The chirality of this structure is physically defined by the helical intensity distribution, with its most essential feature being the capacity to directly confer mechanical chiral manipulation capabilities to the optical field. This approach not only provides a novel physical mechanism for chiral-selective optical manipulation and nano-assembly but also represents a fundamental paradigm shift from passive detection of chirality to active creation and control of chirality.

**Table 1:** Evolution and classification of three types of chiral structured light

Chirality category	Core principle and implementation	Main applications and features	References
Traditional chiral light	**Principle:** relies on the global, static helicity of the light field, such as the rotating polarization vector of circularly polarized light or the helical wavefront of a vortex beam.	**Applications:** traditional circular dichroism spectroscopy, chiral molecule recognition^12,15^.	^50,51^
	**Implementation:** light fields with spatially uniform chiral characteristics, e.g., circularly polarized light, Laguerre-Gaussian beams.	**Features:** a standard and well-established technique; however, the signal is inherently weak due to the scale mismatch between the wavelength of light and molecular size.	
Local chiral light	**Principle:** constructs a local, time-evolving chiral trajectory of the electric field vector by controlling the spatiotemporal distribution of polarization (e.g., using customized femtosecond pulses).	**Applications:** ultrafast chiral dynamics detection, Photoelectron Circular Dichroism (PECD)^52–55^, nonlinear chiral recognition.	^13,56^
	**Implementation:** precise superposition of multiple phase-controllable laser beams.	**Features:** achieves femtosecond-level time resolution, enhancing detection sensitivity by several orders of magnitude, but relies on complex phase and polarization modulation.	
Spatiotemporal intensity chiral light	**Principle:** chirality is directly encoded in the three-dimensional helical trajectory of the energy flow (Poynting vector), independent of polarization and phase. Chirality is an intrinsic property of the intensity distribution itself.	**Applications:** intensity-driven, chirality-selective optical manipulation, nanoparticle assembly, topological photonics.	**This work**
	**Implementation:** programmatically controlled non-collinear collision of STOVs to generate STOCs.	**Features:** chirality is physically imprinted on the energy flow trajectory, enabling the exertion of chiral optical forces and torques on achiral objects. It possesses inherent topological robustness, achieving a paradigm shift from detecting chirality to creating and manipulating chirality.	

In this context, we introduce a distinct strategy that shifts chiral encoding to the three-dimensional spatiotemporal organization of intensity and energy flow. Through the programmable non-collinear collision of spatiotemporal wave packets within an *x*–*y*–*t* volume, we engineer dynamic STOCs with tunable volumetric chirality. We begin by defining a localized chiral measure, *h*_***S***_, along with its normalized scalar counterpart, *ℓ*, based on the Poynting vector, serving as a robust criterion for comparing the strength and sign of chirality. Employing phase-resolved femtosecond tomography, we successfully map the three-dimensional intensity field and track the evolution of singularity lines, directly observing their generation, dynamics, and reconnection. Furthermore, we uncover programmable scaling relations among coil winding number, pitch, and orbital angular momentum density. Crucially, we demonstrate that this volumetric chiral density directly correlates with the capacity to exert mechanical torques, establishing a deterministic physical mechanism for chiral-selective interactions. This work not only introduces intensity-encoded chirality as a new degree of freedom in volumetric topological photonics but also provides a versatile platform for investigating ultrafast topological dynamics and designing sophisticated time-variant optical force landscapes.

## Results

### Generation of secondary vortices from collinear STOV collisions

When two STOV pulses, *E*_1_ and *E*_2_, carrying charges *ℓ*_1_ and *ℓ*_2_, collide in both space and time, their superposition *E*_total_ = *E*_1_ + *E*_2_ can exhibit new phase singularities SV_1_ that go beyond those found in a single beam. Secondary vortices arise at points where the total field amplitude vanishes, and the phase becomes undefined. Geometrically, these singularities correspond to the intersection of the real and imaginary components of the field on sign-changing surfaces (see in Supplementary S1). In other words, if the real part of *E*_total_ changes sign on one surface, and the imaginary part similarly changes sign on another, their intersection forms a trajectory of zero intensity and undefined phase—a topological singularity.

**Fig. 1:**
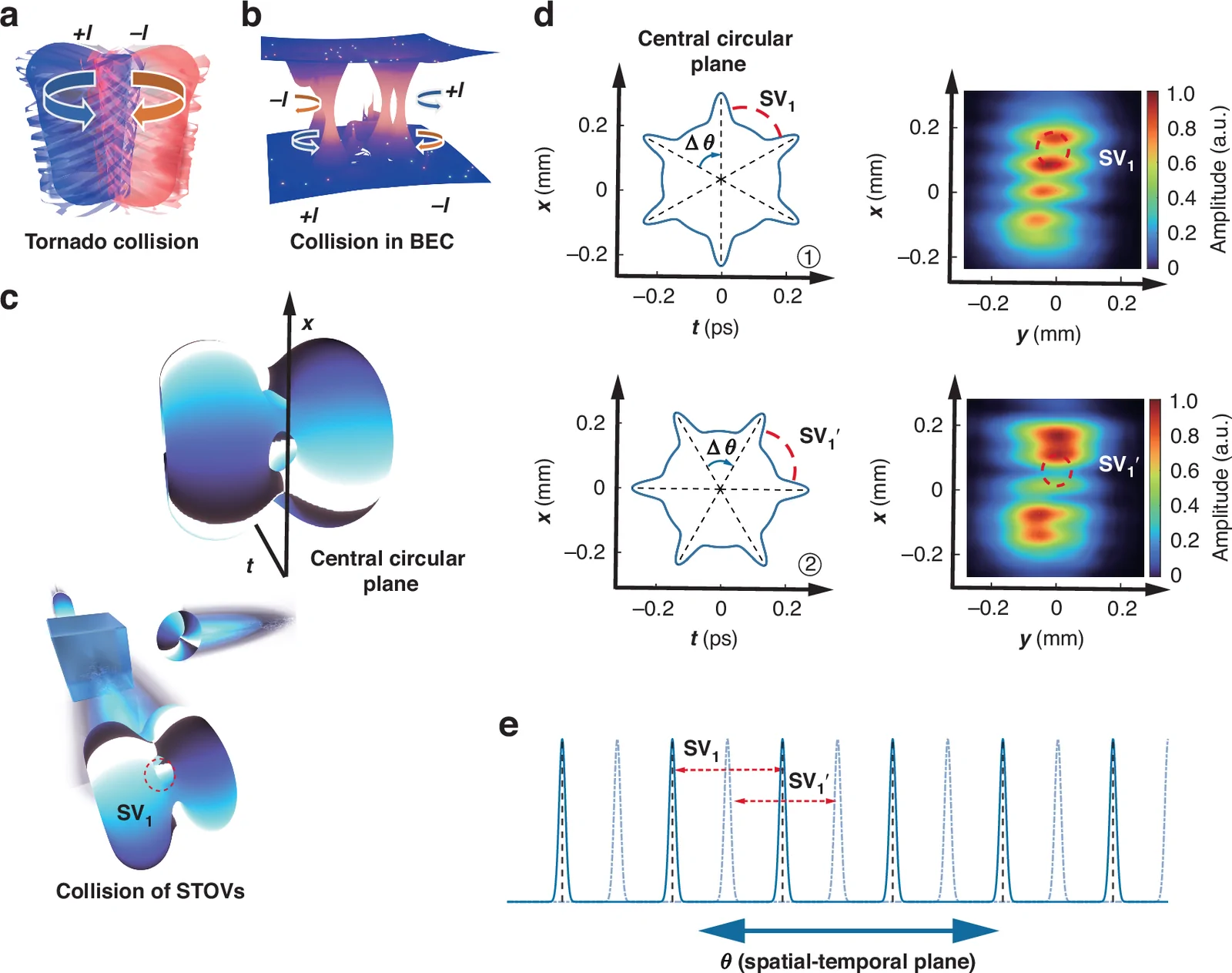
Schematic illustrations of vortex collision phenomena in different contexts. **a** Collision of tornadoes, **b** collision in Bose-Einstein condensates (BECs)^57,58^, and **c** symmetric collision of two STOVs with opposite topological charges (*ℓ* = ±3), illustrating the formation of a secondary vortex array. **d** Geometrical arrangement of the central circular plane with an angular shift Δ*θ* and the secondary vortex SV_1_. As the phase difference between the two optical vortices changes, Δ*θ* and SV_1_ rotate to a new position on the spatiotemporal plane. Correspondingly, the positional variation of the SV is observed in the *x*–*y* plane. **e** Temporal intensity profile of the secondary vortices formed during the STOV collision. The figure highlights the fundamental dynamics of vortex interactions across different systems and provides insight into the creation of complex vortex structures in optical fields

This phenomenon is analogous to phenomena in classical and quantum mechanics, as illustrated in Fig. 1a, b. The collision of two vortices effectively creates a network of sign-changing nodes within the three-dimensional spatiotemporal volume. Crucially, the number of secondary vortices is set by the difference in input charges, *N* = ∣*ℓ*_2_ − *ℓ*_1_∣, with each core carrying a unit topological charge. For example, when STOV pulses with *ℓ*_1_ = +3 and *ℓ*_2_ = −3 collide, *N* = 6 secondary vortices are generated (see Fig. 1c). In the spatiotemporal plane (for instance, a slice through the *x*–*t* plane of the pulse overlap, as shown in Fig. 1c, d), the azimuthal angles of the total field undergo *N* distinct 2*π* phase jumps, resulting in *N* intensity dips. These dips are equidistant in the spatiotemporal plane, spaced by Δ*θ*, forming a comb-like structure in the spatiotemporal domain, see Fig. 1e. This structure is coupled onto the spatiotemporal plane, exhibiting a hollow, periodic, comb-like pattern which we term the Spatiotemporal Hollow Comb (STHC), in analogy with an optical frequency comb.

The offset, defined as the relative phase difference between the two STOV beams, plays a critical role in modulating the secondary vortices. This offset has a similar effect to the concept of offset in traditional optical frequency combs, as shown in Fig. 1e, where it causes a shift in the comb teeth. Each incremental change in Δ*θ* produces a measurable rotation of the entire vortex lattice in the *x*–*t* plane and a concomitant periodic modulation of the integrated *x*–*y* intensity. In our experiments (as in the two configurations depicted in Fig. 1d), we held the initial phase of one STOV fixed, varied that of the other, and recorded a pronounced topological displacement. The phase-offset control provides remarkable tunability, enabling us to shape secondary-vortex arrays into a variety of dynamically changing configurations^40–42^. Adjustments to the phase on a subwavelength scale result in macroscopic lattice rotations, revealing a potent method for tailoring structured light on ultrafast timescales and for directing light-matter interactions with extraordinary precision.

To better convey the ensuing analysis, the electromagnetic field of a STOV is defined as follows^24^:

1 $$\begin{array}{l}\tilde{E}={E}_{0}{A}_{0}{[{(\xi /{w}_{\xi })}^{2}+{(x/{w}_{x})}^{2}]}^{| \ell | /2}\\ \quad \times \exp [-({x}^{2}/{w}_{x}^{2}+{y}^{2}/{w}_{y}^{2}+{\xi }^{2}/{w}_{\xi }^{2})]\\\quad \times \exp [i(-\ell {\varphi }_{{\rm{st}}}+{\omega }_{0}t-{k}_{0}z+\phi )]\end{array}$$

Here *ξ* = *ct* − *z* is a longitudinal coordinate local to the pulse, *c* denotes the group velocity of light, *E*_0_ is the peak electric field strength, *ϕ* is an additional phase of the vortex beam, *A*_0_ is a normalization constant, and *w*_*ξ*_ and *w*_*x*_ represent the spatial scale widths. For a circular spatial profile, we assume *w*_*x*_ = *w*_*y*_ = *r*, where *r* denotes the beam waist radius. ${\varphi }_{{\rm{st}}}={\tan }^{-1}\left(\frac{x{w}_{\xi }}{\xi {w}_{x}}\right)$ is the azimuthal angle in the space–time plane (or *x*–*ξ* plane), and *ω*_0_ and *k*_0_ are the center angular frequency and wave number, respectively. The spatiotemporal trajectory of the singularity, formed by the interference of two STOVs, is governed by two factors. First, it is confined to a surface defined by the condition ∣*E*_1_∣ = ∣*E*_2_∣. Second, its specific directional orientation on that surface is determined by the phase relationship $\arg ({E}_{1})-\arg ({E}_{2})=(2k+1)\pi$.

The surfaces resulting from the overlap of two STOVs satisfy the following condition:

2 $$\left[\left\vert {\ell }_{1}\right\vert \ln (u)-2u\right]-\left[\left\vert {\ell }_{2}\right\vert \ln (v)-2v\right]=2{(y+d)}^{2}/{w}_{{y}_{1}}^{2}-2{(y-d)}^{2}/{w}_{{y}_{2}}^{2}$$

where $u=\frac{{x}^{2}}{{{w}_{x1}}^{2}}+\frac{{\xi }^{2}}{{{w}_{\xi 1}}^{2}}$, $v=\frac{{x}^{2}}{{{w}_{x2}}^{2}}+\frac{{\xi }^{2}}{{{w}_{\xi 2}}^{2}}$, and *ξ* = *ct* − *z*.

Furthermore, the formation of secondary vortices requires the fulfillment of the phase sign-changing condition. A STOV possesses a spatial coordinate and a temporal coordinate that describe its spatiotemporal helical phase. The azimuthal angle of the collision between two STOVs in the spatiotemporal plane (e.g., the *x*–*t* plane) can be expressed as:

3 $${\varphi }_{{\rm{st}}}=\frac{(2n+1)\pi -({\phi }_{1}-{\phi }_{2})}{{\ell }_{2}-{\ell }_{1}}$$

where *N* = ∣*ℓ*_1_ − *ℓ*_2_∣ describes the number of secondary vortices. This equation describes the position of the intensity dips in the wave packet at the same *y*-coordinate for both STOVs. In fact, when there is no phase difference between the two STOVs along the *y*-direction, the overlapping region will give rise to well-defined intensity minima at corresponding positions on the *x*–*t* plane. In these surfaces, we lock the phase singularities, observing their distribution in both time and space.

**Fig. 2:**
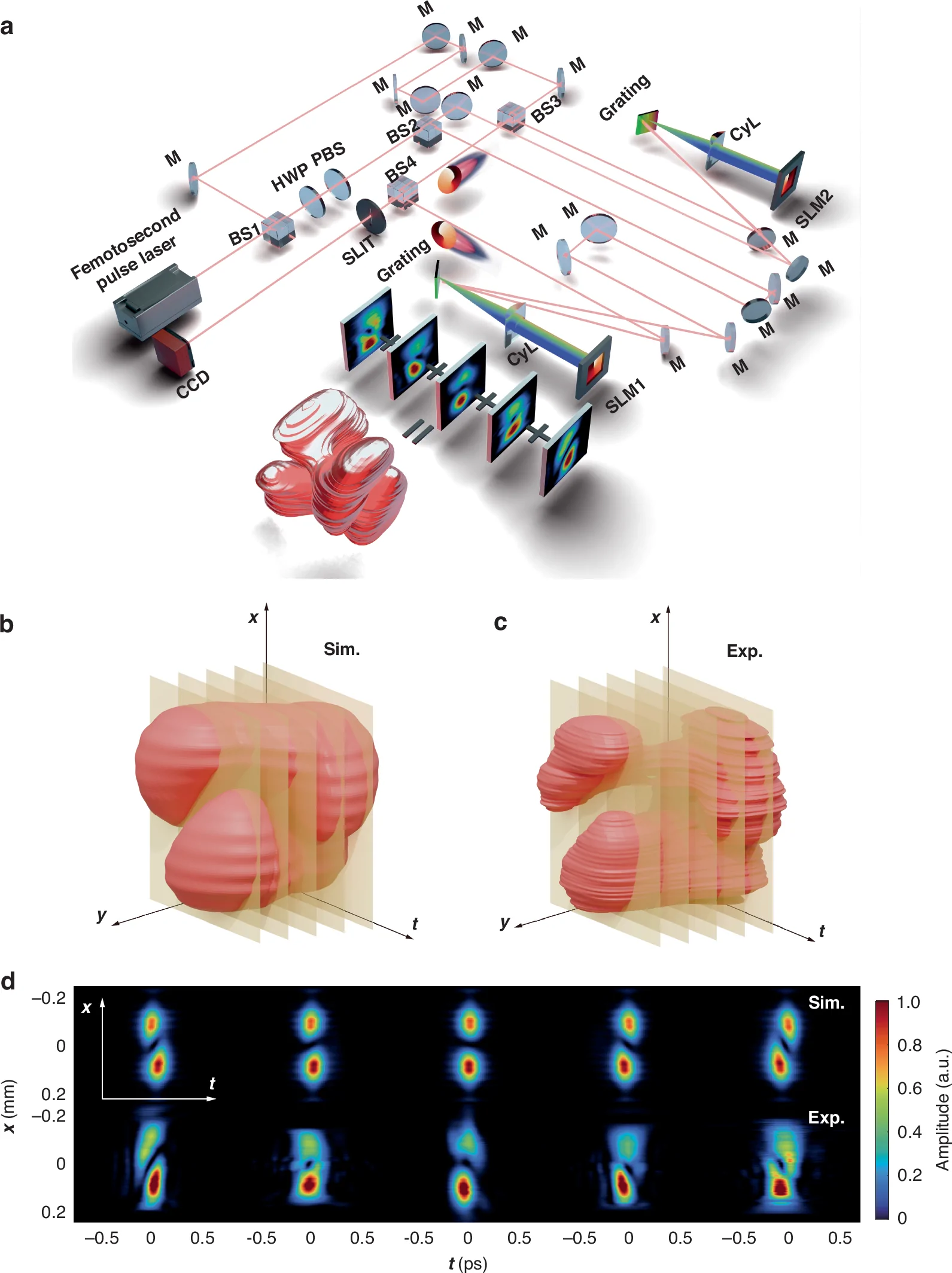
Generation and characterization of secondary vortices from STOV collisions. **a** Experimental setup for generating colliding STOVs. Parameters: Femtosecond pulse laser: 1030 nm, 200 kHz, 170 fs; HWP: Half-wave plate; PBS: Polarization beam splitter; BS: Beam splitter; M: All Mirrors HR @ 1030 nm; 2D pulse shaper: Grating, CyL (Cylinder Lens), SLM (Spatial Light Modulator). **b**, **c** Measured and simulated 3D spatiotemporal field distributions resulting from the collision of a vortex-antivortex pair (*ℓ*_1_ = + 1, *ℓ*_2_ = − 1). **d** Comparison of reconstructed field slices from experiment and simulation at different slit positions

Figure 2a reveals the generation of secondary vortices during the collision of a symmetric topological charge double-STOV pair (*ℓ*_1_ = −1, *ℓ*_2_ = +1). In the experiment, we use a laser with a central wavelength of 1030 nm and a pulse duration of 170 fs. STOVs generated via SLM phase mask encoding in a dual-channel 4*f* pulse shaper are controlled by the helical phase topological charge loaded onto SLM1/SLM2 (Holoeye GAEA-2). Spatiotemporal slices were reconstructed from the interferograms generated between a reference Gaussian beam and spatially resolved sections of the beam, which were sampled using a one-dimensional scanning slit. The intensity between the two STOV beams varies with the slit position. The spectral information of the double STOV exhibits variations at different spatial positions. The slices at different positions reveal the evolution of the vortex and the location of the corresponding secondary vortex. Using spatio-spectral interferometric measurements, we reconstruct the spatiotemporal domain, achieving the reconstruction of the dynamic evolution of the superposition mode between the *ℓ*_1_ = −1 and *ℓ*_2_ = +1 topological charge beams as a function of their relative phase. Figure 2b, c shows the reconstructed three-dimensional spatiotemporal field distribution of the secondary vortex resulting from the collision of a vortex-antivortex pair (*ℓ*_1_ = −1, *ℓ*_2_ = +1). Figure 2d shows the reconstructed intensity distribution in the *x*–*t* plane, obtained from different spatial slices. The experimental setup and reconstruction process are described in more detail in the “Methods and materials” section.

**Fig. 3:**
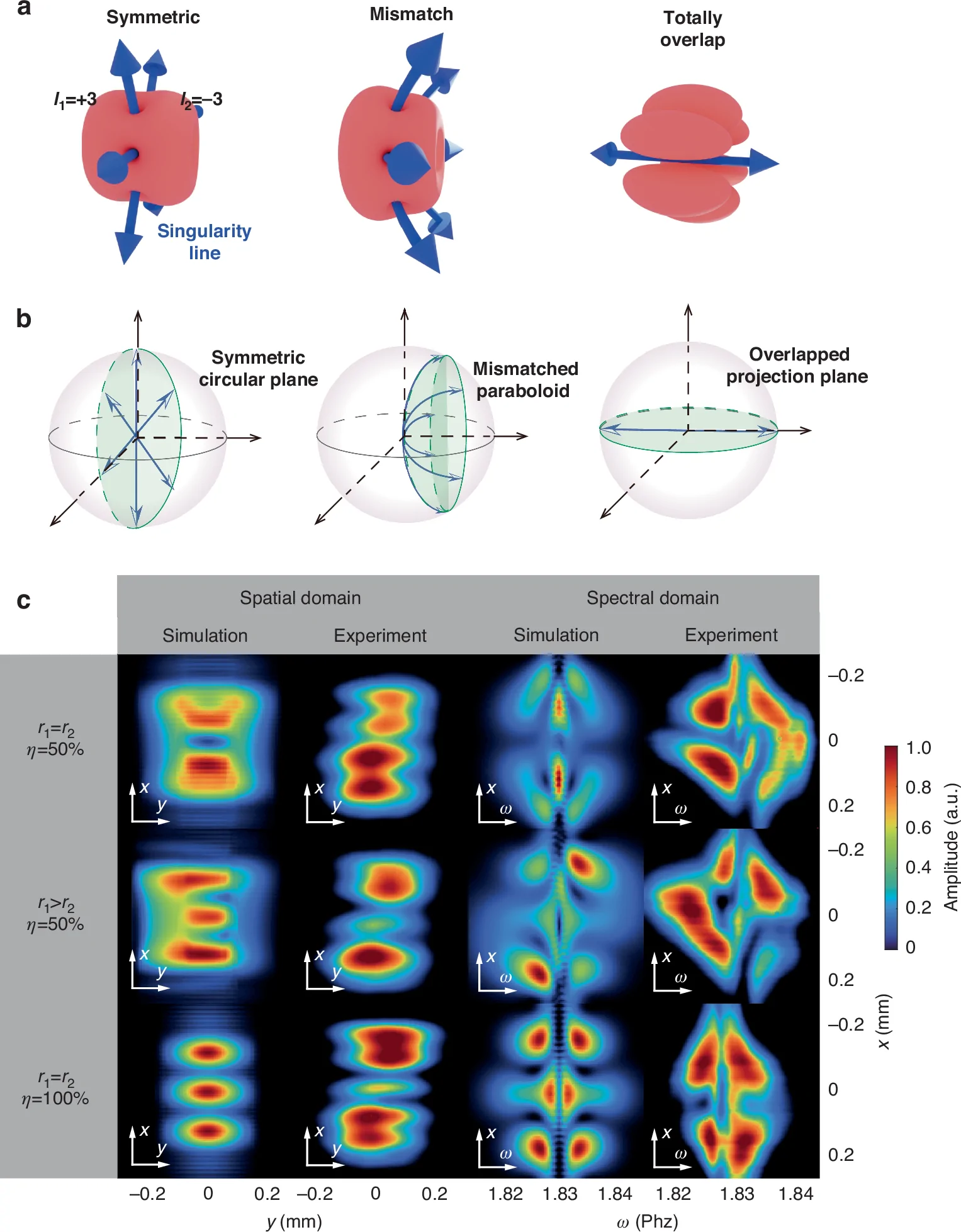
Three distinct situations of collision of STOVs. **a** Schematic representation of the collision between two STOVs with topological charges *ℓ*_1_ = 3 and *ℓ*_2_ = − 3, illustrating the singularity line. **b** Polar plots showing the angular positions of the topological singularities under different conditions. **c** Simulation and experimental results in the spatial domain (left) and spectral domain (right), showing the evolution of secondary vortices under different overlap conditions. The first row shows equal beam waist radius and 50% overlap, resulting in symmetric secondary vortices. The second row shows mismatched beam waist radius and intensity ratios. The third row shows 100% overlap, leading to a petal-shaped interference pattern

By tuning the overlap *η* and beam waist *r*, we steer the phase-singularity trajectories and define three collision regimes: symmetric, mismatched, and fully overlapping. In the case of symmetric interference, where the topological charges of the two pulses are opposite (*ℓ*_1_ = +3 and *ℓ*_2_ = −3), six secondary phase singularities appear at the center of the overlap, forming a symmetric vortex array. These secondary vortices are confined to the *x*–*t* plane (as shown in the left panels of Fig. 3a, b), with the vortex trajectories lying in the plane of collision. When the parameters are mismatched, such as differences in beam waist radius *r*, a radial phase gradient is created. This causes the secondary singularity trajectory to bend toward the smaller spot region, resulting in curved phase singularity lines as the intensity gradient drives the secondary vortices toward the smaller focal area. The secondary vortices therefore escape a single-plane confinement and fan out through the full three-dimensional field (see in Supplementary S1), with the coordinate planes largely spared, as illustrated in the central panels of Fig. 3a, b. Parameter mismatch thus elevates the collision’s complexity, making vortex motion fully three-dimensional and far less predictable. When there is perfect overlap (*η* = 100%), the two pulses generate identical vortices that give rise to a six-lobed structure. This configuration results from the angular interference of the vortices, analogous to the phenomenon observed in superposition states. Here, the secondary vortices are primarily oriented along the *y*-axis, as illustrated at the right side of Fig. 3a, b, thereby establishing a distinct pattern aligned with the beam axis.

In our investigation of STOV collisions, we found that analyzing the interactions in the spectral domain offers significant advantages. Figure 3c showcases our experimental results, where the spectral patterns reveal highly distinct and unambiguous signatures for each of the three collision conditions. This remarkable clarity stems from the inherent stability of the STOVs’ spectral representation. The core structural information encoded in the relative phases of the frequency components is largely preserved. This intrinsic invariance means the spectral domain provides a robust and faithful fingerprint of the interaction, regardless of propagation distance^43,44^. Consequently, the spectral domain serves as an ideal platform for quantitative analysis and the differentiation of subtle physical phenomena. This is powerfully demonstrated by the excellent agreement between our experimental measurements and theoretical simulations, which validates the high precision and reliability of our approach.

### Spatiotemporal optical coil and intensity-induced chirality

More interestingly, when two STOV beams are oriented at a certain propagation angle, they give rise to a coil-like structure. This small collision angle can be approximated as one STOV pulse acquiring a linear phase gradient along the transverse direction (e.g., *y*-axis) relative to the other. This introduces an additional *y*-dependent term to their relative phase difference: Δ*ϕ*_*y*_ = *k*_*y*_*y*, where *k*_*y*_ is the phase variation rate proportional to the collision angle. The total phase difference thus becomes a function of *y*: Δ*Φ*(*y*) = (*ℓ*_2_ − *ℓ*_1_)*φ*_st_ + (*ϕ*_1_ − *ϕ*_2_) + *k*_*y*_*y*. Substituting this phase difference into the destructive interference condition Δ*Φ*(*y*) = (2*n* + 1)*π* yields an equation coupling the spatiotemporal angle *φ*_st_ and spatial coordinate *y*:

4 $$y=\frac{1}{{k}_{y}}\left(({\ell }_{1}-{\ell }_{2}){\varphi }_{{\rm{st}}}+(2n+1)\pi -({\phi }_{1}-{\phi }_{2})\right)$$

The equation describes a three-dimensional helix (or multi-stranded helix). It reveals that the trajectory of secondary vortices in the 3D spatiotemporal space (*x*, *y*, *ξ*) no longer lies within a single plane but instead forms a helical structure—termed a STOC. The two primary geometric properties of this optical coil—the number of helical arms and the pitch—are directly governed by the initial collision parameters. The number of distinct helical arms is determined by the topological charge difference, *N* = ∣*ℓ*_2_ − *ℓ*_1_∣, corresponding precisely to the number of secondary vortices, generated in the collinear case. Meanwhile, the coil’s pitch, which defines its longitudinal period, is dependent on both the collision angle and the topological charge difference. The pitch is inversely proportional to the transverse wavevector component *k*_*y*_, meaning that a larger collision angle produces a tighter, more densely wound coil. Conversely, the pitch is directly proportional to the topological charge difference (*ℓ*_2_ − *ℓ*_1_), indicating that a greater charge difference results in a more stretched, elongated helical structure. This phenomenon closely resembles the coil structure produced by electrons in vortex light fields, where the chiral nature of the coil is manifested in the intensity distribution, rather than solely relying on phase chirality. Since three-dimensional structured light fields possess rich degrees of freedom, when the directions of the two colliding STOVs are not collinear, the additional phase difference is introduced in the overlapping regions of different spatiotemporal positions. This phase difference results in an interference structure that forms a grid-like pattern, both in the spatiotemporal plane and the spatial plane.

**Fig. 4:**
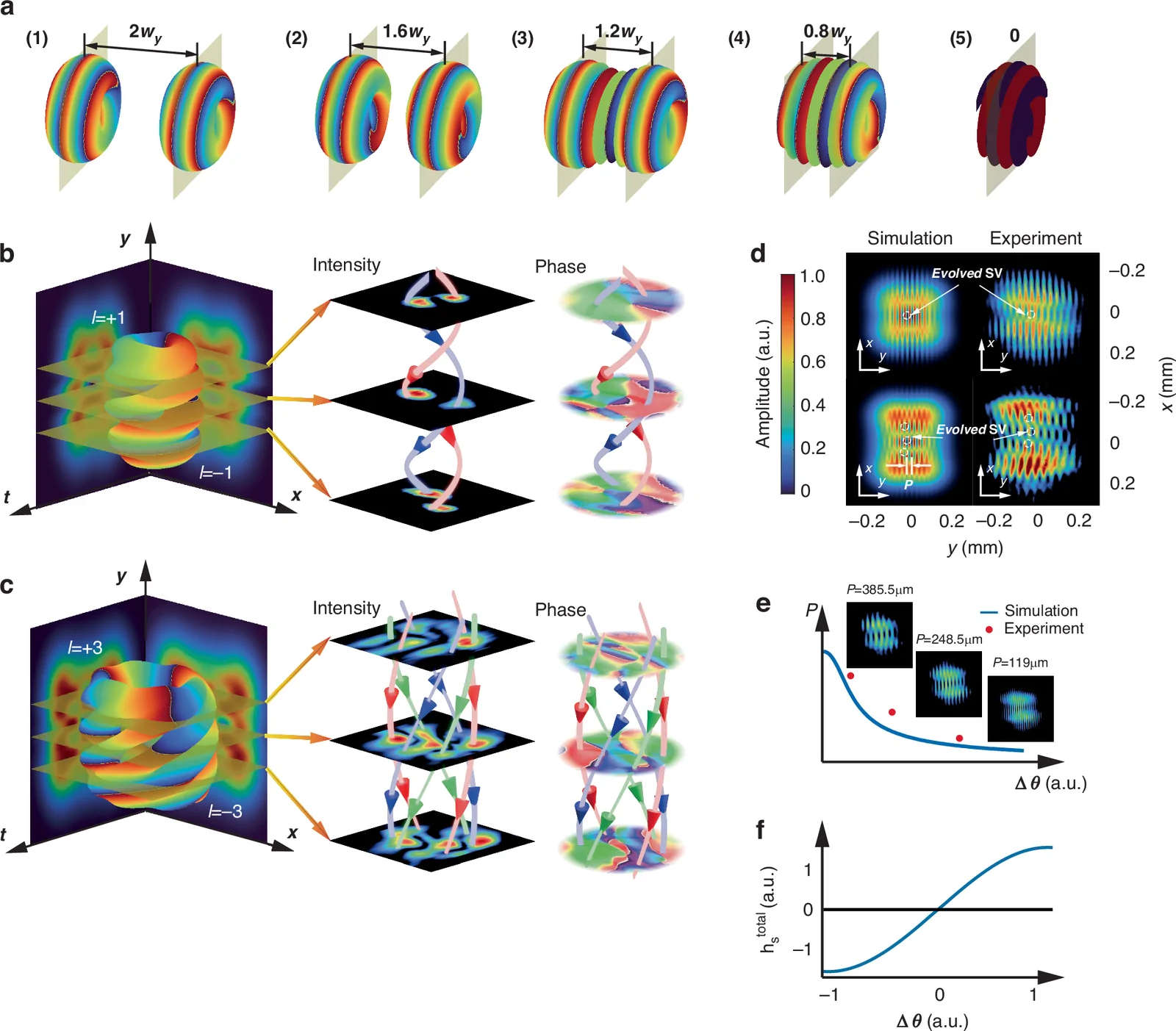
Schematic diagram and experimental results of STOC. **a** Formation of an STOC through the collision of two non-collinear STOVs. Panels (1)–(5) illustrate this process as the initial separation between the STOVs progressively decreases. **b** STOC from *ℓ* = ±1 STOVs, showing rotating intensity and phase distributions in transverse cross-sections. **c** STOC from *ℓ* = ±3 STOVs, exhibiting a different helical rotation with more intertwined helices than the *ℓ* = ±1 case. **d** Spatially integrated intensity patterns for *ℓ* = ±1 (top) and *ℓ* = ±3 (bottom) STOCs, revealing clear forked fringes in simulations and experiments. **e** The STOC’s pitch ***P***, i.e., fringe density varying with the angular separation between colliding STOVs, as shown in the graph and inset images. **f** Variation of *h*_***S***_ with the angle between the two STOVs. The positive and negative signs indicate opposite chiral tendencies

In Fig. 4a, we show a schematic diagram of the collision process between two STOVs at an angle. The two STOVs are slightly non-collinear, intersecting at a small crossing angle of 1^∘^, and thereby generating a phase gradient along the *y*-direction. Figure 4b, c shows the STOC formed by two STOVs with topological charges of ±1 and ±3, respectively. The resulting central STOC behaves like a multi-arm spiral: the number of helical arms scales linearly with ∣*ℓ*_1_ − *ℓ*_2_∣, producing a double helix for the ±1 pair and a six-arm helix for the ±3 pair (Fig. 4b, c). Unlike the STHC in Fig. 1, where helicity appears mainly in 2-D projections such as *x*–*y* or *y*–*t*—the present coil spins throughout the full *x*–*y*–*t* volume.

To visualize this STOC, adjustable slits are positioned at three different *y*-coordinates, facilitating tomographic slicing of the spatiotemporal field (Fig. 4d). A moderate slit width is selected to preserve the interferometric signal-to-noise ratio: this choice precisely resolves the double helix for the ±1 scenario, while occasional arm overlap in the ±3 scenario suggests a more complex structure than can be captured by any individual 2D plane. Projecting the field onto the spatial *x*–*y* plane (Fig. 4e) unveils a distinctive fringe lattice. Horizontal discontinuities encode the ∣*ℓ*_1_ − *ℓ*_2_∣ rhythm of the helical arms, whereas the vertical fringe spacing is inversely proportional to the collision angle between the two STOVs, as quantified in Fig. 4f.

Conventional optical chirality arises from polarization or phase and is often characterized by conserved quantities such as Lipkin’s Zilch^45,46^, defined as:

5 $$C=\frac{{\varepsilon }_{0}}{2}\tilde{{\boldsymbol{E}}}\cdot (\nabla \times \tilde{{\boldsymbol{E}}})+\frac{1}{2{\mu }_{0}}\tilde{{\boldsymbol{B}}}\cdot (\nabla \times \tilde{{\boldsymbol{B}}})$$

where ***E*** is the electric field and ***B*** is the magnetic induction. In contrast to this, our investigation focuses on the chirality inherent to the optical intensity distribution itself. When the field intensity is arranged helically, the energy distribution and energy flow (Poynting vector ***S***, ${\boldsymbol{S}}=\frac{1}{{\mu }_{0}}({\boldsymbol{E}}\times {\boldsymbol{B}})$, *μ*_0_ is the permeability of free space) become inextricably coupled, forming a directed field structure that is non-superimposable on its mirror image. Drawing an analogy with helicity in fluid dynamics, we introduce the energy-flow helicity density to quantify this structural property:

6 $${h}_{{\boldsymbol{S}}}={\boldsymbol{S}}\cdot (\nabla \times {\boldsymbol{S}})$$

This quantity, *h*_***S***_, directly measures the helical topology of the energy flow. Unlike conventional doughnut-shaped vortex beams, where the energy flux curl is typically orthogonal to the flow direction, yielding a vanishing helicity density (*h*_***S***_ ≈ 0), the non-collinear collision in STOCs induces a braiding of energy flow. This results in a non-zero *h*_***S***_, serving as a robust parameter to distinguish this 3D intensity-twisted topology from conventional vortices. This interference-based approach transforms an abstract topological attribute from phase space into a tangible, designable, and engineerable field property. As related research indicates, the essence of chirality can originate from the field’s direct shaping of a particle’s spacetime trajectory. Our helical light field is a physical realization of this concept: it is not only a mathematically rigorous pseudoscalar but also constitutes a geometric driving force that can be directly sensed and responded to by particles. To quantify the global properties of this light field, we can integrate *h*_***S***_ over the spatial domain to obtain ${h}_{{\boldsymbol{S}}}^{\,\mathrm{total}\,}$. By varying the magnitude and sign of the angle between the two STOVs, the chirality of the helical light field can be readily controlled, as shown in Fig. 4f. The pseudoscalar nature of *h*_***S***_ dictates the selection rules for chiral light-matter interactions. A primary consequence is that a first-order response requires a matching pseudoscalar parameter in the interacting material. This provides a direct experimental test: a hallmark of a chirality-dominated effect is the sign reversal of the response signal upon mirror inversion of the field’s structure. Furthermore, since *h*_***S***_ is invariant under time-reversal, it can mediate conservative optical forces and torques, enabling the precise manipulation of particles. The local entanglement of energy flow can therefore not only induce measurable absorption differences in chiral particles but also exert deterministic conservative forces and controllable torques.

**Fig. 5:**
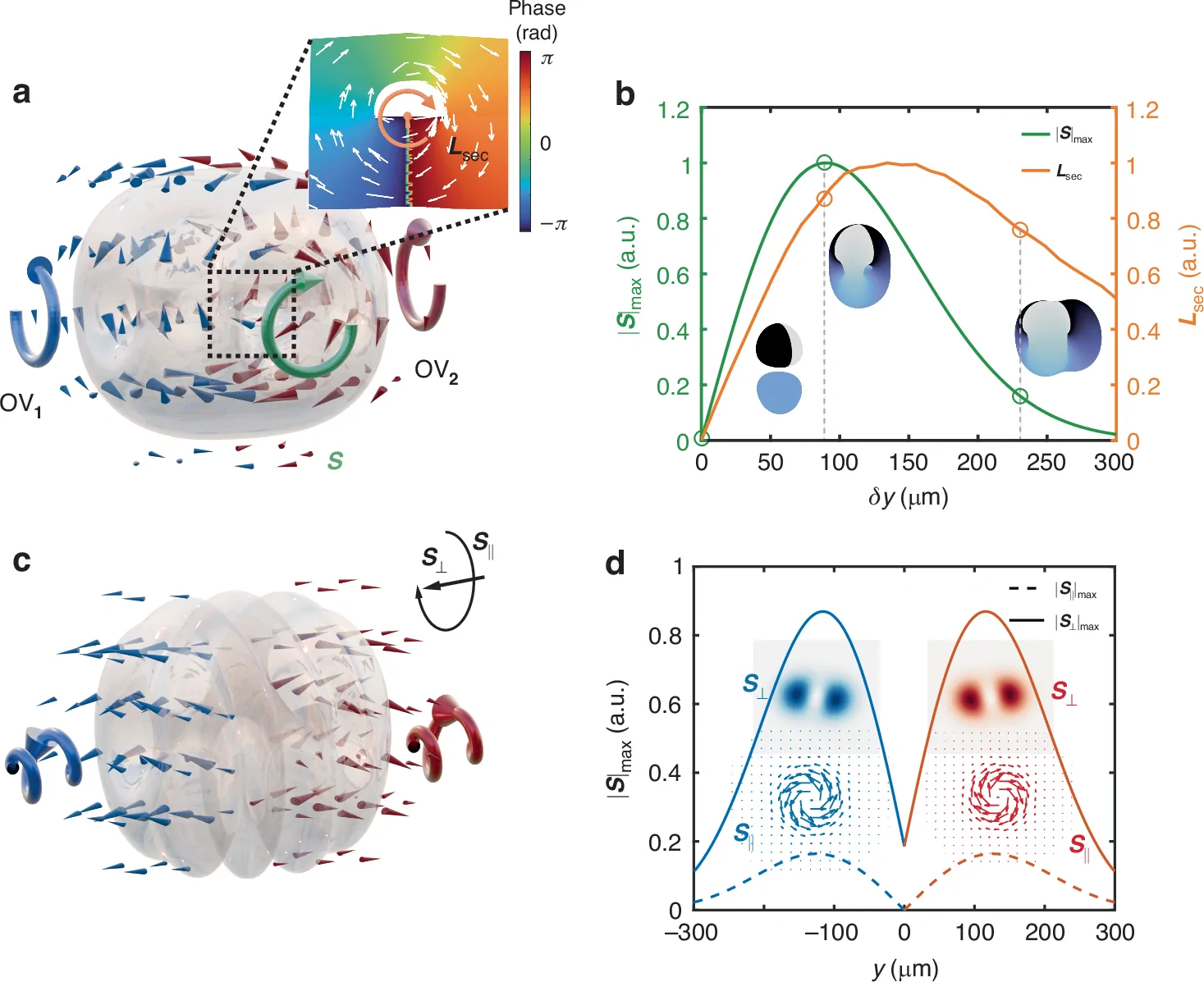
Energy flow characteristics in the collision structure formed by ±1 STOV. **a** Energy flow within a ±1 STOV structure, highlighting Poynting Vector ${\boldsymbol{S}}$ and local circulation around total angular momentum ${{\boldsymbol{L}}}_{\sec }$. The energy flow ${\boldsymbol{S}}$ forms isolated local loops circulating around secondary vortices, representing localized angular momentum. **b** Normalized maximum energy flow $| {\boldsymbol{S}}{| }_{\max }$ on this plane and total angular momentum ${{\boldsymbol{L}}}_{\sec }$ versus vortex separation *δy* = 2*d*, with insets showing corresponding flow patterns. **c** Visualization of the energy flow’s decomposition within the collision structure of a ±1 STOV pair (1^∘^ crossing angle), showing how the isolated local loops from (**a**) are twisted and reconnected into a global helical energy flow by separating the Poynting vector into components parallel (***S***_∥_) and perpendicular (***S***_⊥_) to the *x*–*t* plane. **d** Profile of the maximum magnitudes of ∣***S***_∥_∣ and ∣***S***_⊥_∣ along the transverse *y*-axis. The insets depict how the orientation of the energy flow components varies across different transverse positions

In sharp contrast to classical chiral structured light, whose handedness is engineered via spin angular momentum or phase, the chirality here is inscribed directly into the intensity distribution. Put differently, the helicity stems from the three-dimensional torsion of the energy flow rather than from phase or polarization alone. This makes the phenomenon conceptually closer to the current-density-defined chirality of an electronic coil. Physically, the collinear collision (Fig. 5a) creates a series of isolated energy-flow loops, where the local angular momentum is confined around each secondary vortex. When a crossing angle is introduced (Fig. 5c), the resulting phase gradient twists these localized circulations, reconnecting the isolated loops into a continuous helical energy flow that spans the entire wave packet. Increasing the collision angle compresses the helical pitch and thereby boosts the local chiral density, affording new freedom for enantio-selective excitation and high-dimensional information encoding. Driven solely by intensity, this fully STOC establishes a new paradigm for optical chirality—combining the programmability of STOV topology with volumetric richness unattainable in conventional two-dimensional structured light, and opening unprecedented avenues in chiral photonics and beyond.

The physical relevance of this intensity chirality is profound. As established in the literature^47^, the mechanical response of a chiral particle is mediated by its magnetoelectric polarizability, giving rise to an enantio-selective force. Crucially, it can be derived that the component of this force along the direction of energy flow is directly determined by our proposed local chiral density, *h*_***S***_. This establishes a direct physical link from the field’s intensity landscape to the mechanical transfer of chirality, highlighting *h*_***S***_ as a parameter for controlling the chiral interaction and manipulation of particles (see in Supplementary S2).

We investigate the maximum energy flux intensity, $| {\boldsymbol{S}}{| }_{\max }$, in the bisecting plane perpendicular to the initial vortex separation. Simulations reveal $| {\boldsymbol{S}}{| }_{\max }$ depends on this initial separation, suggesting a controllable mechanism for tuning the resultant optical field’s energy landscape. Structured light’s capacity to exert forces/torques on nanoparticles is linked to its momentum and angular momentum. Secondary vortices, formed during collision, are expected to have unique manipulative capabilities, as shown in Fig. 5a. To quantify this, we examine the optical angular momentum density, ***l***(***r***):

7 $${\boldsymbol{l}}={\boldsymbol{r}}\times \frac{{\boldsymbol{S}}}{{c}^{2}}={\boldsymbol{r}}\times \frac{{\boldsymbol{E}}\times {\boldsymbol{B}}}{{\mu }_{0}{c}^{2}}$$

where ***r*** is the position vector in three-dimensional space. To assess a secondary vortex’s specific influence, we compute its total angular momentum, ${{\boldsymbol{L}}}_{\sec }$, within a strategically chosen cylindrical volume ${V}_{\sec }$ (The complete analysis methods for energy flow and angular momentum are described in Supplementary S3). This volume’s axis aligns with the secondary vortex line, and its radial boundary is defined by local intensity maxima confining the vortex. This ensures ${{\boldsymbol{L}}}_{\sec }$ reflects the secondary vortex’s properties relevant for particle interaction.

The total orbital angular momentum ${{\boldsymbol{L}}}_{\sec }$ is obtained by integrating the angular momentum density, ***l***, over the volume ${V}_{\sec }$. The magnitude and orientation of ${{\boldsymbol{L}}}_{\sec }$ quantify the torque the secondary vortex region can exert, offering insights into its efficacy for particle manipulation. Systematic characterization of energy flux and angular momentum properties versus initial collision parameters (e.g., inter-vortex distance, relative topological charges), as demonstrated in Fig. 5b, advances understanding of vortex interactions and novel optical topologies, informing optical trap design and light-based manipulation techniques.

For the spatiotemporal wave packet formed by the non-collinear collision of STOVs, the internal energy flow exhibits a complex structure, comprising not only a forward-propagating energy flux but also a transverse circulation associated with orbital angular momentum. To separate and quantify these components, we perform an orthogonal decomposition of ***S***. As illustrated in Fig. 5c, the total energy flow is decomposed into a propagation component parallel to the spatiotemporal collision plane (the *x*–*y* plane), denoted ***S***_∥_, and a transverse circulating component perpendicular to this plane, denoted ***S***_⊥_. Here ***S***_∥_ primarily contributes to the overall forward motion of the wave packet, while ***S***_⊥_ directly embodies the intrinsic helicity and twist of the optical field and thus serves as the physical root of the intensity-encoded chirality.

The quantitative plot in Fig. 5d further highlights the intrinsic features of these components. A noteworthy property is their antisymmetry: because the constituent STOVs in the vortex pair rotate in opposite senses relative to the crossing angle, both ***S***_∥_ and ***S***_⊥_ reverse direction at positions symmetric about the center (±*y*). Consequently, both components exhibit a double-peaked intensity profile with vanishing magnitude at the geometric center (*y* = 0), originating from the destructive interference inherent to the singularity generation. Crucially, although the energy-flow vectors are reversed at symmetric positions, the computed local chiral density *h*_***S***_ remains identical there, robustly characterizing the field’s chirality as a pseudoscalar. At the geometric center (*y* = 0), the symmetric contributions destructively interfere, producing a pronounced suppression of both components. Together, these observations indicate that the STOC field’s chirality is fundamentally a dynamic property with a complex spatiotemporal texture in its intensity.

## Discussion and conclusion

Our experiments elevate the study of vortex reconfiguration from schematic two-dimensional pictures to a genuinely three-dimensional, tunable reality. By colliding counter-rotating STOVs, we observe the generation of secondary vortices. The trajectories of these singularities knit an STHC on the central plane, while remaining freely steerable in all three spatial directions through the choice of topological charge, energy balance, and—crucially—the relative phase offset. A comparative analysis with perfect spatiotemporal optical vortices (PSTOVs)^48,49^ is also presented in Supplementary S4. Given their charge-independent geometry, PSTOVs offer an ideal solution to eliminate the radial mismatch inherent in conventional vortex collisions, thereby enabling the synthesis of perfectly symmetric cylindrical coils. The same platform unveils previously unreported STOCs, a chiral structure whose chirality is encoded directly in the energy distribution. This extends the taxonomy of chiral light beyond conventional spin- or phase-based manifestations and offers a new handle for controlling volumetric angular momentum flow. These observations open a rich landscape for future inquiry. The secondary vortex hollow comb, with its offset-dependent drift, suggests a route toward programmable spatiotemporal lattices whose evolution in dispersive or nonlinear media may reveal either soliton-like preservation or, conversely, the controlled topological melting of singularity arrays. Besides, the coil’s chirality is intrinsic to its intensity, which invites investigations into whether this strong-field chirality can survive the nonlinear process. How its behavior in high-order harmonic generation differs from other chiral structures, or if it can selectively address enantiomers in ways inaccessible to conventional circularly polarized light. Extending the scheme to multi-STOV collisions or embedding it within structured dielectrics and quantum materials could entwine orbital and spin degrees of freedom into higher-dimensional entangled states. Furthermore, the ability to prescribe vortex orientation at will foreshadows optically driven torque landscapes tailored for chiral nanomechanics.

In summary, we experimentally demonstrate a novel method for constructing tunable chiral optical fields in three-dimensional spatiotemporal domains. By precisely colliding STOVs, we successfully generate dynamic, inherently chiral helical structures termed STOCs, whose chirality is directly encoded in the light field’s intensity distribution rather than relying on conventional polarization or phase. Our work demonstrates a method for constructing intensity-chiral optical fields, establishes a powerful platform for ultrafast chiral light-matter interactions, topological photonics, and advanced optical nanomanipulation.

## Methods and materials

Figure 2 depicts the experimental setup for colliding dual STOVs. The system comprises a generation module and a characterization module. A high-stability femtosecond laser source based on Yb-doped gain media delivers pulses at a central wavelength of 1030 ± 0.5 nm with 170 fs duration and a 200 kHz repetition rate. An ultrafast beam splitter (BS1) transmits 60% of the input power to the generation module and reflects the remaining 40% to the reference arm for the characterization module.

Within the generation module, the incident beam is split by BS2 into two arms, each coupled into identical 4*f* pulse shapers. Each shaper employs a 1200 lines mm^−1^ blazed diffraction grating and a cylindrical lens (*f* = 200 mm) in a strictly confocal Fourier-transform geometry. The grating and cylindrical lens separation is precisely matched to the 200 mm focal length to achieve linear spatial-spectral mapping. The frequency components of the pulse are angularly dispersed via first-order diffraction by the blazed grating. Reflective liquid-crystal spatial light modulators (SLM1 and SLM2; Holoeye GAEA-2, 3840 × 2160 pixels, 3.74 μm pixel pitch) are positioned at the Fourier plane and programmed with phase/amplitude mask functions. Using SLM1 and SLM2, we control both the topological charge of the generated STOV pulses and the relative phase Δ*θ* between the two STOVs. The characterization module utilizes an unmodulated Gaussian beam split directly from the laser output as the reference arm. This reference beam is delayed by a time *δt* relative to the modulated dual-STOV beam using a precision air-bearing translation stage for optical path difference adjustment. It enables spatio-spectral interferometric measurements of the shaped pulses and provides independent characterization employing spectral phase interferometry via spatial-axis shearing scan for electric field reconstruction. The beams from both modules are spatially mode-matched at the combining beam splitter (BS4) and undergo collinear interference before entering the core grating of the spectral analysis module. The resulting high dynamic range (>16 bit) spectral interference patterns are acquired by a CCD spectrometer. The complex electric field envelope is then reconstructed from the data using a fast Fourier transform-based phase retrieval algorithm.

To reconstruct the complex amplitude of the synthesized optical fields, we employed Fourier Transform Spectral Interferometry, a technique based on spectral interferometry. In this method, the signal field *E*_s_ is collinearly interfered with a time-delayed (Δ*t*) reference pulse *E*_r_, generating a spatio-spectral interferogram *I*(*x*, *ω*). The time delay introduces a spectral fringe pattern carrier, $\cos (\omega \Delta t)$, which encodes the phase difference $\Delta \phi (\omega )=\arg ({E}_{{\rm{s}}})-\arg ({E}_{{\rm{r}}})$ into the intensity modulation.

The retrieval procedure, illustrated in detail in Supplementary S5, involves a Fourier analysis of the recorded interferograms. We first perform a Fourier transform along the spatial axis to separate the phase-carrying cross-correlation terms as sidebands in the spatial-frequency domain. By isolating one of these sidebands and comparing it to a calibration measurement (from reference-reference interference), we can robustly extract both the spectral phase and amplitude of the signal field. A final inverse Fourier transform along the frequency axis yields the full spatiotemporal field, *E*(*x*, *t*), revealing its amplitude profile and characteristic helical phase structure.

## Supplementary information

Supplementary Information: Generation of Spatiotemporal Optical Coil with Controllable Transverse Intensity Chirality

## Data Availability

The data that support the findings of this study are available from the corresponding author upon reasonable request.
