# Supplementary material for: Generation of spatiotemporal optical coil with controllable transverse intensity chirality
Source: Light Sci Appl. 2026 Nov 9;15:397. doi: 10.1038/s41377-026-02432-0 (PMC13745593; doi:10.1038/s41377-026-02432-0)
Supplement: Supplementary file 1 [file 41377_2026_2432_MOESM1_ESM.pdf]

Supplementary Information: Generation of Spatiotemporal Optical Coil with Controllable Transverse Intensity Chirality

Dana·Jiashaner,[1,*] Xingyuan Zhang,[2,*] Huiming Wang,[3,*] Zhirong Tao,[4] Qian Cao,[1] Dawei Liu,[1] Daijun Luo,[1] Lu Yang,[2] Kaili Geng,[2] Yonghang Tai,[5] Xinhua Xie,[6] Zhensheng Tao,[7] Md Sabbir Ahsan,[8] Guangyu Fan,[1,†] and Qiwen Zhan[1,9]

[1]*School of Optical-Electrical and Computer Engineering, University of Shanghai for Science and Technology, Shanghai 200093, China*
[2]*Zhejiang Key Laboratory of 3D Micro/Nano Fabrication and Characterization, Westlake Institute for Optoelectronics, Fuyang, Hangzhou, Zhejiang 311400, China*
[3]*Shanghai Jiao Tong University, Shanghai 201100, China*
[4]*Karlsruhe Institute of Technology, Karlsruhe, Germany*
[5]*Department of Physics and Electronic, Yunnan Normal University, Kunming, Yunnan 650000, China*
[6]*Photon Science Center, Paul Scherrer Institute, 5232 Villigen PSI, Switzerland*
[7]*State Key Laboratory of Surface Physics and Department of Physics, Fudan University, Shanghai 200433, China*
[8]*Fachbereich Physik, Freie Universität Berlin, Arnimallee 14, 14195 Berlin, Germany*
[9]*Zhejiang Key Laboratory of 3D Micro/Nano Fabrication and Characterization, School of Engineering, Westlake University, Hangzhou, Zhejiang 310030, China*

S1. THEORETICAL DESCRIPTION OF SECONDARY VORTICES (SINGULARITY LINES)

The complex amplitude field of a spatiotemporal optical vortex (STOV) pulse can be described as[1]:

$$E(x, y, z, t) = E_0 A_0 \left[\frac{\xi^2}{w_\xi^2} + \frac{x^2}{w_x^2} \right]^{|\ell|/2} \exp\left[-\left(\frac{x^2}{w_x^2} + \frac{y^2}{w_y^2} + \frac{\xi^2}{w_\xi^2} \right) \right] \exp\left[i(-\ell\varphi_{\rm st} + \omega_0 t - k_0 z + \phi) \right] \tag{S1}$$

Here, (x, y, z, t) denotes the space-time coordinates, and $\xi = ct - z$ is the longitudinal coordinate in the co-moving coordinate system, c is the speed of light, E_0 is the electric field peak amplitude, and A_0 is the normalized amplitude. w_x, w_y, w_ξ represent the beam waist sizes in the spatial and temporal dimensions, respectively. Following the convention in the main text and figures, we assume a symmetric spatial profile $w_x = w_y = r$, where r denotes the transverse beam waist radius. Here, $\varphi_{\rm st} = \arctan\left(\frac{x w_\xi}{\xi w_x}\right)$ is the azimuthal angle in the space-time plane (or $x - \xi$ plane), which simplifies to $\varphi_{\rm st} = \tan^{-1}(x/\xi)$ under the assumption $w_x = w_\xi$. ℓ is the topological charge (orbital angular momentum quantum number), and ϕ is the initial phase of the pulse. When two STOV pulses (E_1 and E_2), carrying topological charges ℓ_1 and ℓ_2 respectively, interfere, their superimposed optical field is given by $E_{\rm total} = E_1 + E_2$. In optics, for a phase singularity (vortex) or, more generally, a singularity line, a necessary condition is that the complex amplitude optical field vanishes[2]. This condition can be expressed as:

$$E_{\rm total}(x, y, z, t) = 0 \tag{S2}$$

Since the electric field is complex, this equation is equivalent to the simultaneous vanishing of its real and imaginary parts:

$$\mathrm{Re}\,(E_{\rm total}) = 0 \tag{S3}$$

$$\mathrm{Im}\,(E_{\rm total}) = 0 \tag{S4}$$

The curve formed by the intersection of the set of planes determined by these two equations is the spatial locus of the singular points. From $E_1 + E_2 = 0$, two more intuitive conditions can be directly derived:

1. **Amplitude Condition**: The amplitudes of the two pulses must be equal: $|E_1| = |E_2|$.
2. **Phase Condition**: The phases of the two pulses must differ by an odd multiple of π: $\arg(E_1) - \arg(E_2) = (2n+1)\pi, \quad n \in \mathbb{Z}$.

These two conditions must be simultaneously satisfied at every point along the singularity line, collectively determining the generation, number, and spatiotemporal trajectory of secondary vortices.

* These authors contributed equally to this work.
† gfan@usst.edu.cn

According to the first condition, the equation can be derived as:

$$[|\ell_1|\ln(u) - 2u] - [|\ell_2|\ln(v) - 2v] = 2(y+d)^2/w_{y_1}^2 - 2(y-d)^2/w_{y_2}^2 \tag{S5}$$

where $u = \frac{x^2}{w_{x1}{}^2} + \frac{\xi^2}{w_{\xi 1}{}^2}$, $v = \frac{x^2}{w_{x2}{}^2} + \frac{\xi^2}{w_{\xi 2}{}^2}$, and $\xi = ct - z$. d is half of the distance between the two STOVs in the y-direction. The specific spatial shape determined by the equations depends on the parameters of the two vortex beams. In the main text, we simplify the conditions by assuming $w_x = w_\xi = r, \ell_1 = -\ell_2$ and $w_{y1} = w_{y2}$. Based on these assumptions, we explore several relatively simple cases: 1. When $r_1 = r_2, d \neq 0$, the amplitude matching condition is only satisfied on the central plane between the two beams, given by the simple relation: $y = 0$. 2. When $r_1 \neq r_2, d \neq 0$, the locus of points with equal amplitude forms a spatiotemporal paraboloid, described by the equation: $x^2 + \xi^2 = \frac{r_1^2 r_2^2}{2(r_1^2 - r_2^2)}\left[|\ell|\ln\left(\frac{r_2^2}{r_1^2}\right) - \frac{8yd}{w_y^2}\right]$. 3. When $r_1 = r_2, d = 0$, the amplitude condition is satisfied everywhere in space and time. This condition corresponds to the superposition state of vortex beams mentioned in previous research[3]. The orientation of the secondary vortices is determined by the phase condition:

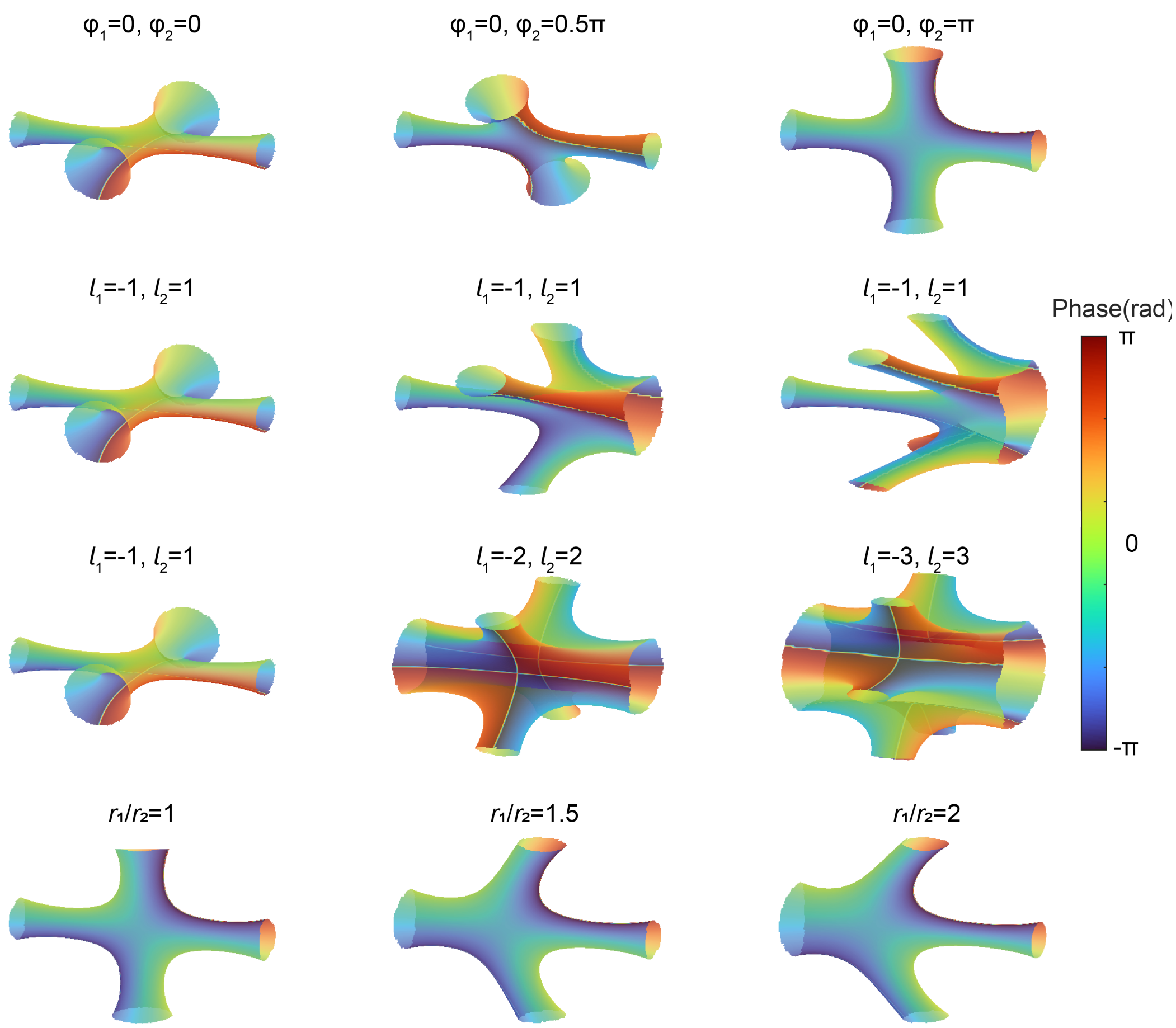

FIG. S1. **Morphological evolution of secondary vortices generated in the collinear collision of two STOV pulses under different parameter conditions.**

$$\Delta\Phi = \arg(E_1) - \arg(E_2) = (-\ell_1\varphi_{st} + \phi_1) - (-\ell_2\varphi_{st} + \phi_2) = (\ell_2 - \ell_1)\varphi_{st} + (\phi_1 - \phi_2) \tag{S6}$$

For two vortex pulses with topological charges ℓ_1 and ℓ_2 and initial phases ϕ_1 and ϕ_2, the phase condition is given by: $\Delta\Phi = (2n+1)\pi$, the azimuthal position φ_{st} of the secondary vortex in the spatiotemporal plane can be solved as:

$$\varphi_{st} = \frac{(2n+1)\pi - (\phi_1 - \phi_2)}{\ell_2 - \ell_1} \tag{S7}$$

From Equation (S7), two key conclusions can be derived regarding the quantity and position of the resulting secondary vortices. Firstly, the number of solutions to the equation within one 2π phase cycle is given by $N = |\ell_2 - \ell_1|$, which directly determines the number of secondary vortices (i.e., the teeth of the Spatiotemporal Hollow Comb, STHC) generated on the central spatiotemporal plane. Furthermore, the angular position φ_{st} of these vortices is directly dependent on the initial phase difference $(\phi_1 - \phi_2)$ between the two pulses. This relationship is significant because precisely controlling this phase difference enables the continuous rotation of the entire spatiotemporal comb structure within the spatiotemporal plane, thus establishing a theoretical foundation for dynamically manipulating these secondary vortices. As shown in Fig. S1, we visually demonstrate precise and deterministic control over the morphology of secondary vortices generated from the collinear collision of two STOV pulses. By systematically isolating and varying key parameters, we can predictably engineer the resulting spatiotemporal structures. This gallery of simulations confirms that these parameters serve as independent handles for the targeted manipulation of secondary vortices, allowing for the precise sculpting of their shape and orientation.

To ensure the reproducibility and validate the experimental findings, three-dimensional numerical simulations were performed based on the linear superposition of complex spatiotemporal wave packets. The simulation framework utilizes the analytical STOV model described in Eq. (S1) as the governing equation to define the complex field $E(x, y, z, t)$ of individual pulses. The initial conditions are strictly matched to the experimental parameters: a central wavelength $\lambda = 1030$ nm, a spatial beam waist $w_\xi = w_x = w_y \approx 300\,\mu$m, and a pulse duration $\tau = 170$ fs. To accurately model the non-collinear interference required for STOC generation, we implemented a spectral-domain phase modulation approach: the individual complex fields were first transformed via a Fast Fourier Transform (FFT) along the temporal axis, modulated by a transverse phase factor $\exp[ik_y y \sin\theta]$ to introduce the specific collision angle θ, and then inverse-transformed to reconstruct the total superimposed field E_{total}. This method ensures the preservation of phase-front curvature and timing jitter accuracy. The 3D singularity trajectories (singularity lines) were subsequently extracted by identifying the volumetric intersections where the conditions $\text{Re}(E_{\text{total}}) = 0$ and $\text{Im}(E_{\text{total}}) = 0$ are simultaneously satisfied. This rigorous numerical procedure confirms the topological consistency between the theoretical predictions and the experimental phase-resolved measurements.

S2. INTENSITY-ENCODED CHIRALITY AND OPTOMECHANICAL MANIPULATION

When two vortex beams are obliquely superposed at a certain angle, the tilted phase manifests the phase chirality, which originally exists in the complex amplitude, in the intensity distribution. This leads to the formation of a spiral structure with a definite handedness, accompanied by the generation and migration of secondary vortices. To transform this qualitative phenomenon into a computable, comparable, and experimentally verifiable description, we introduce a local chirality index, $h_{\boldsymbol{S}}$, in our work. It is sensitive to the energy flow and curl of the field, and can be locally integrated in space to obtain the global intensity chirality.

$$h_{\boldsymbol{S}} = \boldsymbol{S} \cdot (\nabla \times \boldsymbol{S}) \tag{S8}$$

It is important to clarify that $h_{\boldsymbol{S}}$ represents a local helicity density. The corresponding global property of the optical field, $h_{\boldsymbol{S}}^{\text{total}}$, can be obtained by integrating $h_{\boldsymbol{S}}$ over the entire space: $h_{\boldsymbol{S}}^{\text{total}} = \int_{\mathbb{R}^3} h_{\boldsymbol{S}}\, dV$ It is well-established that a conventional vortex beam possesses chirality in its phase structure and, consequently, in the direction of its energy flux. However, due to its inherent symmetry, it does not exhibit chirality in its intensity distribution. For such a standard vortex beam, the local helicity $h_{\boldsymbol{S}}$ is zero. This is because in the absence of a transverse energy flux (i.e., along the y-axis in our discussion), the curl of the energy flux is orthogonal to its direction. Conversely, when two vortex beams are superposed at a slight angle, the resulting transverse component of the energy flux yields a non-zero $h_{\boldsymbol{S}}$. This corresponds to the depiction in Fig. 4 of the main text. On one hand, the presence of a non-zero $h_{\boldsymbol{S}}$ indicates the existence of a helical optical field and, consequently, of intensity chirality. On the other hand, the sign of $h_{\boldsymbol{S}}$ directly reflects the handedness (positive or negative) of this intensity chirality. As elucidated in the review by Mun et al.[4], the mechanical response of a chiral particle is mediated by its coupling to the field via the magnetoelectric polarizability, $\boldsymbol{\alpha}_{\text{c}}$. In the dipole approximation, the time-averaged chiral force is given by:

$$\begin{aligned}\langle \mathbf{F} \rangle = &-\nabla \langle \mathbf{U} \rangle + \frac{\sigma}{c} \langle \mathbf{\Phi}_{\text{u}} \rangle + c \nabla \times [\sigma_{\text{e}} \langle \mathbf{L}_{\text{e}} \rangle + \sigma_{\text{m}} \langle \mathbf{L}_{\text{m}} \rangle] \\ &+ \frac{1}{c} \,\text{Im}(\alpha_{\text{c}}) \nabla \times \langle \mathbf{\Phi}_{\text{u}} \rangle + \frac{c k_0^5}{3\pi} \,\text{Re}(\alpha_{\text{e}} \alpha_{\text{c}}^*) \langle \mathbf{L}_{\text{e}} \rangle \\ &+ \frac{c k_0^5}{3\pi} \,\text{Re}(\alpha_{\text{m}} \alpha_{\text{c}}^*) \langle \mathbf{L}_{\text{m}} \rangle - 4 k_0^2 \,\text{Im}(\alpha_{\text{c}}) \langle \mathbf{\Phi}_{\text{h}} \rangle\end{aligned} \tag{S9}$$

Here, $\mathbf{\Phi}_{\text{u}}(\mathbf{r})$ is the time-averaged Poynting vector, which describes the directional energy flux density of the electromagnetic field: $\mathbf{\Phi}_{\text{u}}(\mathbf{r}) = \frac{1}{2}\,\text{Re}(\tilde{\mathbf{E}} \times \tilde{\mathbf{H}}^*)$. Within this decomposition of the total optical force, the term of particular interest is $\frac{1}{c}\,\text{Im}(\alpha_{\text{c}}) \nabla \times \langle \mathbf{\Phi}_{\text{u}} \rangle$, which is identified as the vortex force. This component originates from the energy-flow vortex and is directly proportional to both the imaginary part of the particle's magnetoelectric polarizability, $\text{Im}(\alpha_{\text{c}})$, and the curl of the time-averaged Poynting vector, $\nabla \times \langle \mathbf{\Phi}_{\text{u}} \rangle$. Here, α_{c} is the magnetoelectric polarizability, which for a chiral sphere of radius R in the quasi-static limit is given by:

$\alpha_c = 4\pi R^3 \frac{3\kappa}{(\varepsilon_r+2)(\mu_r+2)-\kappa^2}$. This specific term is pivotal as it establishes the direct physical link between the topological structure of the energy flow — which our local chirality index h_S is designed to quantify — and the resulting mechanical manipulation of chiral matter.

$$\mathbf{F}_{\text{vortex}}(\mathbf{r}) = \frac{1}{c}\,\mathrm{Im}(\alpha_c)(\nabla \times \boldsymbol{S}) \tag{S10}$$

Consequently, the component of this enantioselective force along the direction of energy flow is locally determined by h_S:

$$\mathbf{F}_{\text{vortex}} \cdot \boldsymbol{S} = \frac{1}{c}\,\mathrm{Im}(\alpha_c)\boldsymbol{S} \cdot (\nabla \times \boldsymbol{S}) = \frac{1}{c}\,\mathrm{Im}[\alpha_c]h_{\boldsymbol{S}} \tag{S11}$$

This reveals that chiral mechanical effects are directly correlated with the topological features of the energy flow, for which h_S is the direct measure. The capacity of the field to directly shape a particle's trajectory is thus supported by a verifiable physical model. The intensity chirality of our helical field, as an intrinsic property of the wavepacket, not only reinforces fundamental physical concepts of chirality but also opens new frontiers in chiral separation, selective photocatalysis, and nanoscale mechanical manipulation.

S3. ANALYSIS METHODS FOR ENERGY FLOW AND ANGULAR MOMENTUM DENSITY

To gain deeper insights into the physical properties of complex three-dimensional structures formed by colliding dual STOV pulses, we conduct detailed analyses of energy transport pathways and spatial distributions of angular momentum. These quantities are critical for revealing the potential of light-matter interactions, including optical trapping forces and optically induced torques. In electromagnetic fields, energy flow is rigorously described by the Poynting vector $\boldsymbol{S}$, defined as:

$$\boldsymbol{S} = \frac{1}{\mu_0}\boldsymbol{E} \times \boldsymbol{B} \tag{S12}$$

where $\boldsymbol{E}$ and $\boldsymbol{B}$ are the electric field intensity and the magnetic flux density, respectively, and μ_0 is the vacuum permeability. The Poynting vector specifies both the direction of energy propagation and the energy flux density. For linearly polarized optical fields, $\boldsymbol{S}$ can be further expressed via the complex amplitude of the electric field $\boldsymbol{E}$[5]:

$$\begin{aligned} \boldsymbol{S} &= \frac{c^2\varepsilon_0}{2\omega}\mathrm{Im}\left(\boldsymbol{E}^* \times (\nabla \times \boldsymbol{E})\right) \\ &= \frac{c^2\varepsilon_0}{2\omega}\mathrm{Im}\left((\nabla \boldsymbol{E}) \cdot \boldsymbol{E}^*\right) + \frac{c^2\varepsilon_0}{4\omega}\nabla \times \mathrm{Im}\left(\boldsymbol{E}^* \times \boldsymbol{E}\right) \end{aligned} \tag{S13}$$

where ε_0 is the vacuum permittivity, and c is the speed of light. The first and second terms represent the orbital and spin angular momentum components, respectively. Our analysis places particular emphasis on the orbital angular momentum (OAM) contribution to the energy flow. By isolating this component from the linear propagation momentum, a circulating energy flux pattern emerges, as depicted in Fig. S2a. We demonstrate this by computing the 3D energy flow structure for a representative case of colliding vortices with topological charges $\ell = \pm 1$ as shown in Fig. S2b. The analysis of various cross-sections further elucidates the intricate energy dynamics and localization around the resultant secondary vortices. Notably, the system is energetically closed with no external dissipation; however, we observe a pronounced energy exchange between the two vortex pulses. The intensity of this interaction is critically governed by their initial separation. By systematically varying this distance, we identified an optimal separation that maximizes the energy flux across the mid-plane. This peak signifies the strongest coupling and the most efficient energy transfer between the interacting vortices. The OAM of an optical field directly embodies its rotational properties. Its density distribution $\boldsymbol{l}$ is defined via the linear momentum density $\boldsymbol{g} = \boldsymbol{S}/c^2$:

$$\boldsymbol{l} = \boldsymbol{r} \times \boldsymbol{g} = \frac{1}{c^2}\boldsymbol{r} \times \boldsymbol{S} \tag{S14}$$

where $\boldsymbol{r}$ is the position vector relative to the rotation center. $\boldsymbol{l}$ describes the local OAM contribution at every point in space. To quantitatively evaluate the potential of a secondary vortex to exert optical torque on microscopic particles, we focus on its total orbital angular momentum. The total OAM $\boldsymbol{L}_{\text{sec}}$ for a single secondary vortex is computed by integrating the OAM density over a specific volume V_{sec}:

$$\boldsymbol{L}_{\text{sec},k} = \int_{V_{\text{sec}}} \boldsymbol{l}(\boldsymbol{r})\,dV \tag{S15}$$

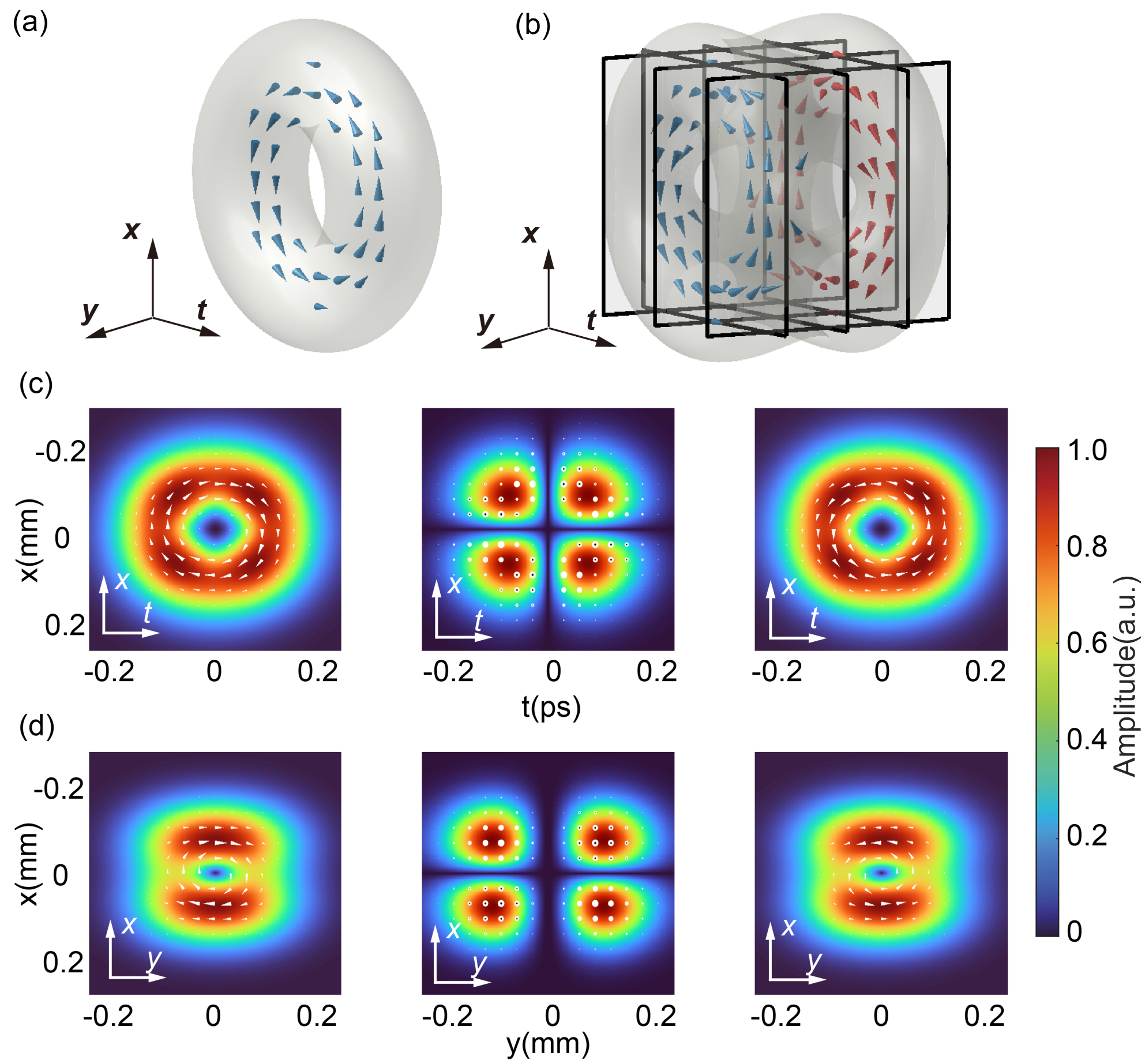

FIG. S2. **Analysis of orbital energy flux density in STOV pulse collision.** **(a)** 3D visualization of the circulating energy flux for a single STOV pulse ($\ell = +1$). **(b)** Energy flux of two colliding STOVs ($\ell = \pm 1$), where black frames indicate the positions of the cross-sectional planes. **(c)** Intensity (color map) and energy flux (white arrows) distributions on the spatio-temporal ($x - t$) plane at three different y-positions. The central panel corresponds to the y=0 plane. **(d)** Corresponding distributions on the spatial ($x - y$) plane at three different time instances. The central panel corresponds to t=0. White arrows indicate the direction of energy flow.

The construction process of the V_{sec} is illustrated in Fig. S3. This integration volume is constructed by sweeping a variable cross-sectional area along the vortex's singularity line. The tangential direction at any given point on the line is indicated by the magenta arrow. This cross-section is defined in the normal plane, with its boundary set at half the radial distance to the first surrounding local intensity peak (schematically outlined by the white closed curve).

The resulting direction and magnitude of the OAM provide a quantitative physical foundation for understanding and designing optical manipulation techniques based on STOV collisions.

S4. COMPARATIVE ANALYSIS WITH COLLISIONS OF PERFECT SPATIOTEMPORAL OPTICAL VORTICES

As we discussed in S1, the dimensions of the annular structure of conventional STOVs, including spatial and temporal diameters are typically strongly correlated with the magnitude of their topological charge $|\ell|$. Consequently, when the topological charge is altered, the orientation of the resulting secondary vortex lines also changes, deviating from the straight lines observed in the ordinary case. It is important to specify that our discussion pertains to a more generalized situation. We note that the generation of perfect spatiotemporal optical vortices (PSTOVs) has been addressed in previous research[6]. Here, we can also incorporate them into the scope of our discussion. A PSTOV can be considered a simplified and purer spatiotemporal vortex

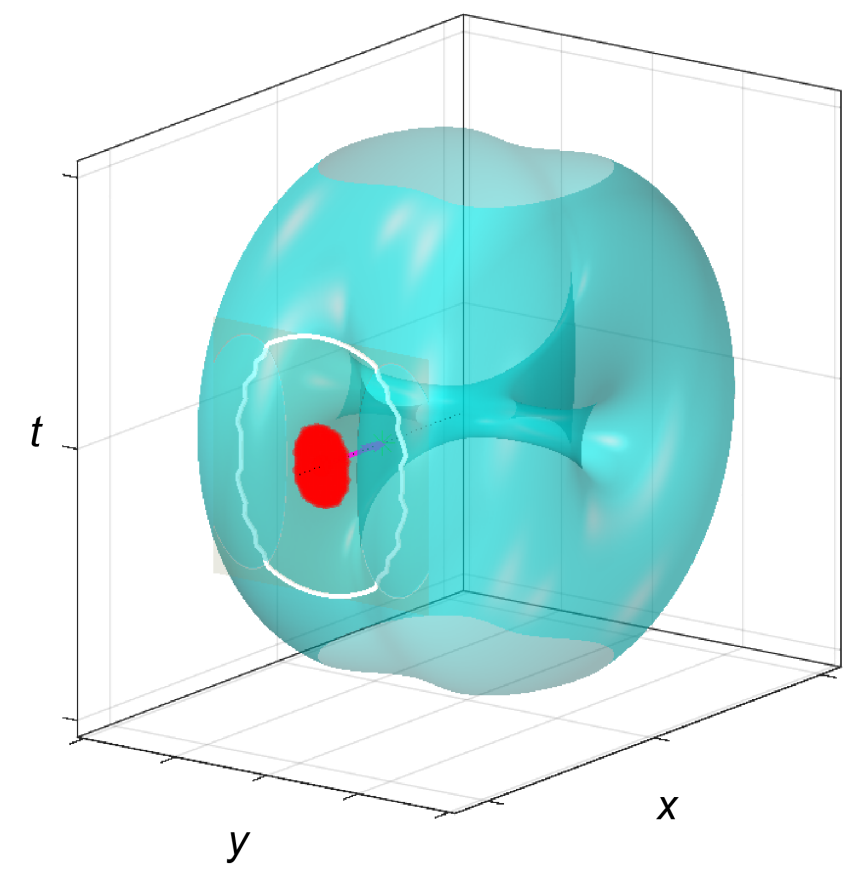

FIG. S3. **Schematic illustration of the construction of the integration volume for calculating the orbital angular momentum (OAM) of a secondary vortex.**

mode, with the defining characteristic that the size of its spatiotemporal ring is independent of its topological charge ℓ. PSTOVs are generated by applying a two-dimensional spatiotemporal Fourier transform to a spectrally engineered Bessel-Gaussian beam in the (k_x, ω) domain. The complex amplitude $\psi(\rho, \phi_{\text{spec}})$ in the spectral domain is expressed as:

$$\begin{aligned} \psi(r,\theta) &= \text{FT}\left\{\psi(\rho,\phi)\right\} \\ &= \frac{1}{2\pi}\int_0^{2\pi}\int_0^{\infty} \exp\left(-\frac{\rho^2}{w_0^2}\right) J_\ell(a\rho) e^{-i\ell\phi} e^{i\rho r\cos(\phi-\theta)} \rho d\rho d\phi \\ &\approx i^{\ell-1}\frac{w_0}{w}\exp\left(-\frac{(r-r_0)^2}{w^2}\right)e^{-i\ell\theta} \end{aligned} \tag{S16}$$

where $(\rho, \phi_{\text{spec}})$ denote polar coordinates in the spectral domain, J_ℓ is the ℓ-th order Bessel function of the first kind, w_0 is the waist radius of the Gaussian envelope, and a is a radial wavevector parameter governing the oscillation frequency of the Bessel function. The ring radius $r_0 = af/k_0$ depends solely on the scaling factor a and the focal length f of the lens implementing the Fourier transform (k_0: central wavenumber). Crucially, r_0 is independent of the topological charge ℓ. This stands in stark contrast to conventional Laguerre-Gaussian-like STOVs, whose ring dimensions grow with $\sqrt{|\ell|}$. The formula above represents a simplified 2D case. In the actual three-dimensional (3D) scenario, the expression should be as follows:

$$E(x,y,z) = i^{\ell-1}\frac{w_0}{w}\exp\left(-\frac{(\sqrt{x^2+\xi^2}-r_0)^2}{w^2}\right)\exp\left(-\frac{y^2}{w_y^2}\right)\exp(-i\ell\varphi_{\text{st}})\exp(i\phi_{\text{offset}}) \tag{S17}$$

where $\xi = c * t - z$, $\varphi_{\text{st}} = \arctan(x/\xi)$. The exponential term can be treated as a phase term: $i^{\ell-1} = (e^{i\pi/2})^{\ell-1} = e^{i(\ell-1)\pi/2}$. By rearranging the amplitude condition, we obtain the following relationship for the positions of the secondary vortices:

$$\frac{(r-r_2)^2}{w_2^2} - \frac{(r-r_1)^2}{w_1^2} + \frac{(y-d)^2}{w_{y2}^2} - \frac{(y+d)^2}{w_{y1}^2} = \ln\left(\frac{w_{02}w_1}{w_2 w_{01}}\right) \tag{S18}$$

Similarly, the phase condition for destructive interference is simplified to:

$$\varphi_{\text{st}} = \frac{(2n+1)\pi - (\phi_{\text{offset1}} - \phi_{\text{offset2}}) + (\ell_2 - \ell_1)\frac{\pi}{2}}{\ell_2 - \ell_1} \tag{S19}$$

From these two equations, we can conclude that the number of vortices generated from the collision of perfect STOVs is still determined by the difference in their topological charges, $|\ell_2 - \ell_1|$. The specific positions of these vortices are also influenced by their respective topological charges through the phase condition. For the amplitude condition, we assume that the w_y and the spectral-domain waist radius are the same for both colliding vortices ($w_{y1} = w_{y2} = w_y$, $w_{01} = w_{02}$), and discuss a few simplified cases:

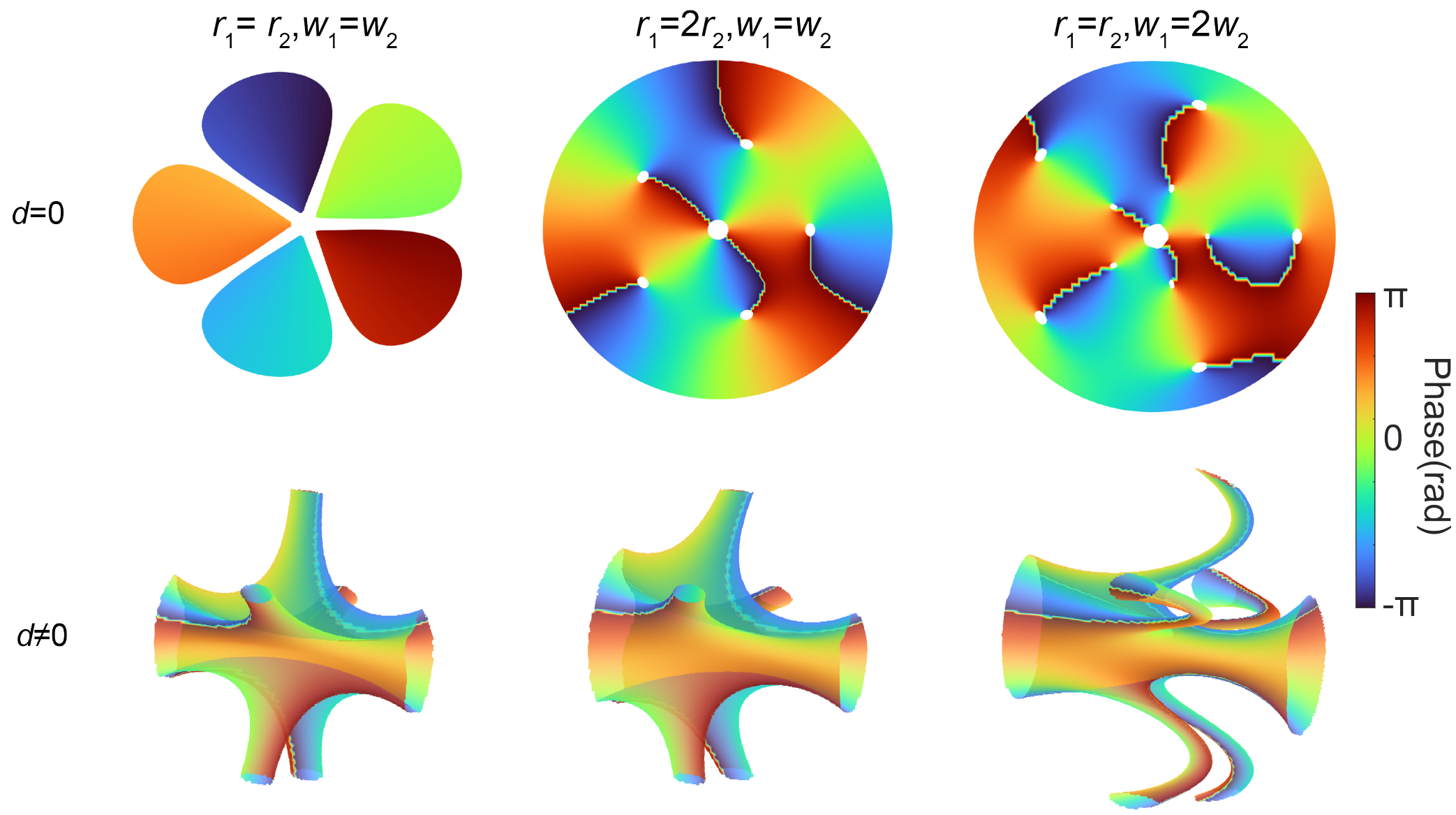

FIG. S4. **Morphology of secondary vortices from PSTOV collisions under different initial parameters for collinear (d=0, top row) and non-collinear (d≠0, bottom row) cases.**

1. When all other parameters of the two vortices are identical ($r_{01} = r_{02}$, $w_1 = w_2$, etc.), the amplitude condition yields a solution of $y = 0$. This implies that the resulting vortex lines will lie on the perpendicular bisector plane, a result analogous to that of conventional STOVs. Notably, for the special case where $d = 0$, a petal-like interference pattern emerges due to the direct superposition of the vortices.

2. When the ring thicknesses w are the same but the central radii r_0 are different, the amplitude equation becomes:

$$r(y) = \frac{r_1 + r_2}{2} + \frac{2yd}{r_1 - r_2} \tag{S20}$$

This equation describes a conical surface in space, meaning the secondary vortices will be distributed on this cone. Specifically, when $d = 0$, the equation simplifies to:

$$r(y) = \frac{r_1 + r_2}{2} \tag{S21}$$

In this scenario, the amplitude condition defines a cylindrical surface, leading to the appearance of several new vortices oriented in the same direction as the original ones.

3. When the central radii r_0 are the same, but the ring thicknesses w are different, the equation transforms into:

$$(r - r_0)^2 = \frac{C_0 + \frac{4yd}{w_y^2}}{\frac{1}{w_2^2} - \frac{1}{w_1^2}} \equiv \frac{N(y)}{D} \tag{S22}$$

Here, $C_0 = \ln\left(\frac{w_{02}w_1}{w_2 w_{01}}\right)$. If $d = 0$, we obtain two distinct solutions for the radius:

$$r_\pm = r_0 \pm \sqrt{\frac{C_0}{D}} \tag{S23}$$

This indicates the emergence of two sets of spatiotemporal vortex lines, each corresponding to a different spatiotemporal orbital angular momentum along the y-axis. When $d \neq 0$, these two sets of vortex lines will exhibit a more complex trajectory, showing a quadratic dependence on the y coordinate.

We implemented a numerical model to simulate the collinear collision of two PSTOVs with different topological charges ($\ell_1 = -3$, $\ell_2 = 2$). The corresponding simulation results, depicted in Fig. S4, clearly validate our theoretical predictions. The figure illustrates the morphological evolution of secondary vortices under three distinct parameter sets, with the top row showing the $x - t$ plane view for collinear collisions (d=0), and the bottom row displaying the 3D trajectories of the singularity lines for non-collinear collisions (d≠0). The simulations confirm that the symmetric case ($r_1 = r_2, w_1 = w_2$) produces the expected planar vortex array, while the case of unequal ring radii ($r_1 \neq r_2$) results in a conical vortex surface. Notably, for the case of unequal ring widths ($w_1 \neq w_2$), the inner and outer rings of discrete vortices generated at d=0 merge into a single, highly curved and complex vortex line when d≠0. This topological reconnection corresponds precisely to the point in space where the two independent radial solutions from our theoretical model become degenerate, thereby joining the inner and outer vortex sets. Crucially, the recent experimental realization of PSTOVs [7] validates the feasibility of this theoretical framework. As analyzed in the main text (Fig. 3), constructing multi-arm STOCs using conventional STOVs often results in curved or conical trajectories due to the radius mismatch ($r \propto \sqrt{|\ell|}$). By adopting the PSTOV scheme described here, where the ring radius r_0 is decoupled from ℓ, one can enforce $r_1 = r_2$ even for collisions with distinct topological charges (e.g., $\ell_1 = 1, \ell_2 = -3$). This capability is essential for generating STOCs with perfect cylindrical symmetry and uniform chiral density, providing a robust platform for future applications requiring high-purity topological fields.

S5. EXPERIMENTAL SETUP FOR GENERATING SECONDARY VORTICES AND PHASE RETRIEVAL PROCESS USING FTSI

To achieve precise reconstruction of the complex amplitude of a STOV field, we employ an experimental method based on spectral interferometry. The core principle of this technique is to encode the phase information of the test optical field — not directly recordable by detectors — into a measurable spatio-spectral intensity distribution through interference with a known reference beam. In the experimental setup, the test signal field $E_s(\omega, y)$ and reference field $E_r(\omega, y)$ are superimposed with a time delay Δt. The resulting 2D interferogram intensity $I(\omega, y)$ on a CCD camera is precisely described by:

$$\begin{aligned} I(\omega, y) &= |E_s(\omega, y) + E_r(\omega, y)|^2 \\ &= I_s(\omega, y) + I_r(\omega, y) + E_s(\omega, y)E_r^*(\omega, y) + E_s^*(\omega, y)E_r(\omega, y) \end{aligned} \tag{S24}$$

Here, $I_s = |E_s|^2, I_r = |E_r|^2$ represent the intensities of the signal and reference fields, respectively, which constitute the background DC component of the interferogram. The latter two terms are the cross-interference terms, containing the spectral phase difference $\Delta\phi(\omega) = \arg(E_s) - \arg(E_r)$ that we ultimately need to solve. Expressing the cross-term in cosine form, Equation (S24) becomes:

$$I(\omega) = I_s(\omega) + I_r(\omega) + 2\sqrt{I_s(\omega)I_r(\omega)}\cos(\omega\Delta t - \Delta\phi(\omega)) \tag{S25}$$

In this equation, the time delay Δt introduces a linear spectral phase carrier $\omega\Delta t$. This experiment employs Fourier Transform Spectral Interferometry (FTSI) to accurately reconstruct the complex amplitude of a STOV under test. As shown in Fig. S5, the procedure begins by acquiring a calibration interferogram from the reference beam interfering with itself (a), and a signal interferogram from the interference between the reference and the STOV (b). Both interferograms are first Fourier-transformed along the spatial axis, converting them to the spatial-frequency domain (c, d). This isolates the phase-carrying cross-correlation term as a sideband, separated from the DC background. A band-pass filter, indicated by the yellow lines, is then used to extract the corresponding complex sideband signals from both datasets. The core reconstruction consists of two key operations: First, the spectral phase (f) is retrieved by calculating the complex ratio of the signal sideband to the calibration sideband and taking its argument, a method that effectively cancels common-mode phase errors from the optical path. Second, the spectral amplitude (e) is recovered by normalizing the modulus of the signal sideband. Finally, a Fourier transform is applied to the reconstructed complex spectrum — composed of the amplitude (e) and phase (f) — along the frequency axis to retrieve the complete spatio-temporal field of the STOV, revealing its intensity profile (g) and its characteristic helical phase structure (h).

[1] Jhajj, N. et al. Spatiotemporal optical vortices. *Physical Review X* **6**, 031037 (2016).

[2] Soskin, M. S., Gorshkov, V. N., Vasnetsov, M. V., Malos, J. T. & Heckenberg, N. R. Topological charge and angular momentum of light beams carrying optical vortices. *Physical Review A* **56**, 4064-4075 (1997).

[3] Woźniak, P. et al. Tighter spots of light with superposed orbital-angular-momentum beams. *Physical Review A* **94**, 021803 (2016).

[4] Genet, C. Chiral light–chiral matter interactions: an optical force perspective. *ACS Photonics* **9**, 319-332 (2022).

[5] Allen, L., Padgett, M. & Babiker, M. *IV The Orbital Angular Momentum of Light*, 291-372 (Elsevier, 1999).

[6] Fan, H. H. et al. Perfect spatiotemporal optical vortices. *Photonics Research* **13**, 1776-1782 (2025).

[7] Zhang, S. S. et al. Controllable perfect spatiotemporal optical vortices. Print at `https://arxiv.org/abs/2501.10055` (2025).

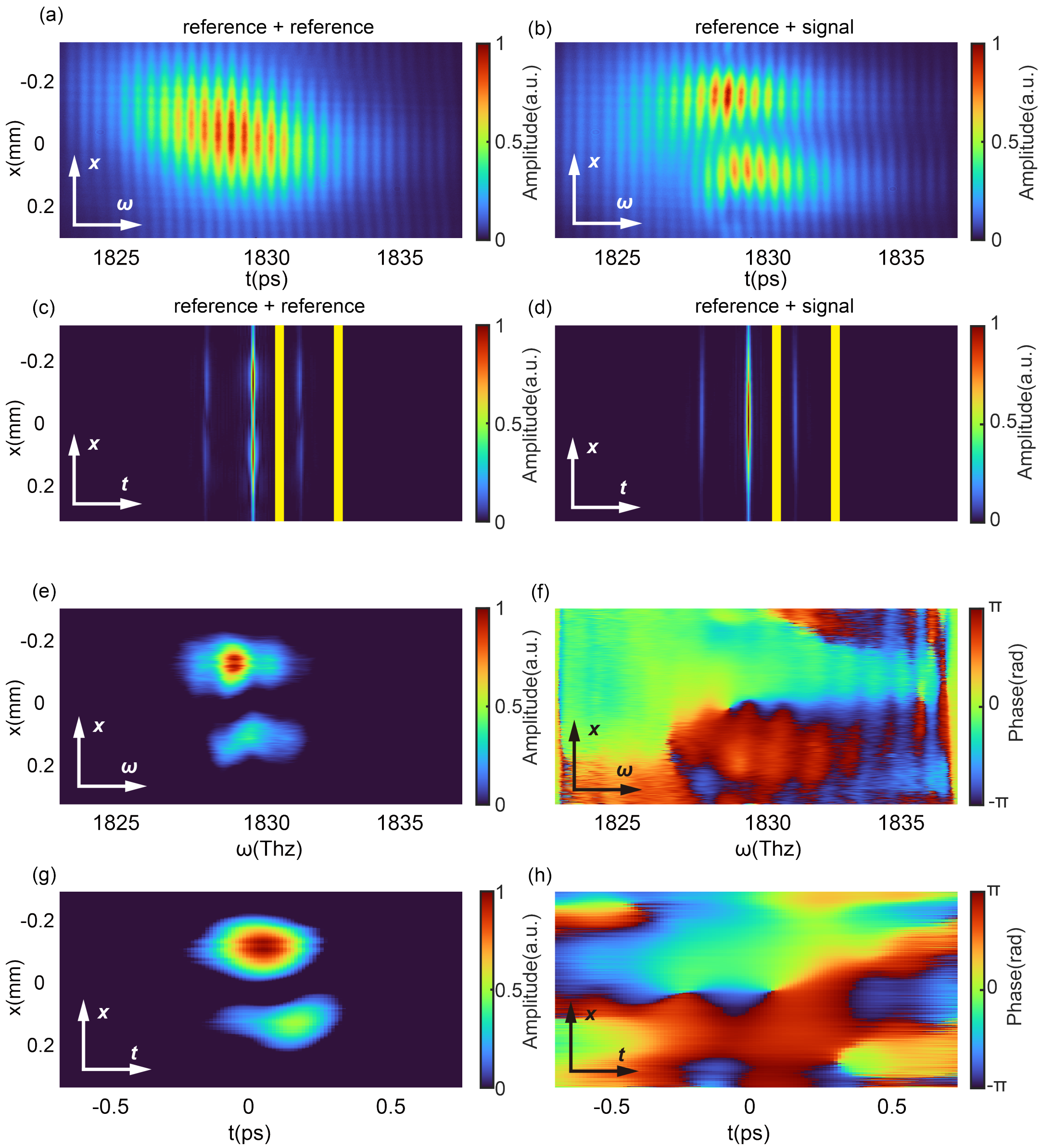

FIG. S5. **Experimental phase retrieval of the STOV complex field using Fourier Transform Spectral Interferometry (FTSI). (a, b)** Raw spatio-spectral ($x-\omega$) interferograms for the calibration (reference + reference) and signal (reference + signal) measurements, respectively. **(c, d)** Spatially Fourier-transformed data of the interferograms shown in (a) and (b). The yellow lines indicate the band-pass filter used to isolate the cross-correlation sideband containing the phase information. **(e, f)** Reconstructed spectral amplitude and spectral phase of the STOV pulse, retrieved from the filtered sidebands. **(g, h)** Final reconstructed spatio-temporal ($x-t$) amplitude and phase profile of the STOV, obtained by applying an inverse Fourier transform along the frequency axis to the complex spectrum composed of (e) and (f). The helical structure in (h) confirms the vortex nature of the retrieved field.
